# Co-Dispersion Delivery Systems with Solubilizing Carriers Improving the Solubility and Permeability of Cannabinoids (Cannabidiol, Cannabidiolic Acid, and Cannabichromene) from *Cannabis sativa* (Henola Variety) Inflorescences

**DOI:** 10.3390/pharmaceutics15092280

**Published:** 2023-09-04

**Authors:** Anna Stasiłowicz-Krzemień, Piotr Szulc, Judyta Cielecka-Piontek

**Affiliations:** 1 Department of Pharmacognosy and Biomaterials, Faculty of Pharmacy, Poznan University of Medical Sciences, Rokietnicka 3, 60-806 Poznan, Poland; astasilowicz@ump.edu.pl; 2 Department of Agronomy, Poznań University of Life Sciences, Dojazd 11, 60-632 Poznan, Poland; piotr.szulc@up.poznan.pl; 3 Department of Pharmacology and Phytochemistry, Institute of Natural Fibres and Medicinal Plants, Wojska Polskiego 71b, 60-630 Poznan, Poland

**Keywords:** cannabidiol, cannabidiolic acid, cannabichromene, cannabis, solubility, permeability

## Abstract

Cannabinoids: cannabidiol (CBD), cannabidiolic acid (CBDA), and cannabichromene (CBC) are lipophilic compounds with limited water solubility, resulting in challenges related to their bioavailability and therapeutic efficacy upon oral administration. To overcome these limitations, we developed co-dispersion cannabinoid delivery systems with the biopolymer polyvinyl caprolactam-polyvinyl acetate-polyethylene glycol (Soluplus) and magnesium aluminometasilicate (Neusilin US2) to improve solubility and permeability. Recognizing the potential therapeutic benefits arising from the entourage effect, we decided to work with an extract instead of isolated cannabinoids. *Cannabis sativa* inflorescences (Henola variety) with a confirming neuroprotective activity were subjected to dynamic supercritical CO_2_ (scCO_2_) extraction and next they were combined with carriers (1:1 mass ratio) to prepare the co-dispersion cannabinoid delivery systems (HiE). In vitro dissolution studies were conducted to evaluate the solubility of CBD, CBDA, and CBC in various media (pH 1.2, 6.8, fasted, and fed state simulated intestinal fluid). The HiE-Soluplus delivery systems consistently demonstrated the highest dissolution rate of cannabinoids. Additionally, HiE-Soluplus exhibited the highest permeability coefficients for cannabinoids in gastrointestinal tract conditions than it was during the permeability studies using model PAMPA GIT. All three cannabinoids exhibited promising blood-brain barrier (BBB) permeability (P_app_ higher than 4.0 × 10^−6^ cm/s), suggesting their potential to effectively cross into the central nervous system. The improved solubility and permeability of cannabinoids from the HiE-Soluplus delivery system hold promise for enhancement in their bioavailability.

## 1. Introduction

*Cannabis sativa* L. is a plant rich in secondary plant metabolites as it contains cannabinoids, terpenes, flavonoids, amino acids, fatty acids, phytosterols, vitamins, and minerals [1]. Cannabis flowers, also known as inflorescences, possess a range of potential medicinal properties such as analgesic, anti-inflammatory, and antiemetic effects [2,3,4]. Additionally, cannabis flowers have shown promise in aiding sleep, stimulating appetite, and modulating neurological conditions like epilepsy [5,6]. Academic research is progressively expanding to explore the medicinal capabilities of cannabis flowers and their constituents.

Cannabinoids, such as tetrahydrocannabinol (THC), cannabidiol (CBD), cannabidiolic acid (CBDA), cannabigerol (CBG), or cannabichromene (CBC) are lipophilic constituents of *Cannabis sativa* L. that are poorly soluble in water (2–10 μg/mL) [7], which is the result of their lipophilic nature (log P 6–7) [8]. This is a limitation for cannabinoid oral administration as only dissolved compounds can be absorbed across the gastrointestinal epithelium [9], which results in low bioavailability (THC: 4–12%; CBD: ≈6%) [10,11]. The solubility of a molecule is a key determinant of its gastrointestinal fate and poorly soluble compounds may require formulation strategies, such as micronization, lipid-based formulations, or complexation, to improve their solubility and enhance their oral bioavailability [12,13]. However, findings in the literature in this field focus on work with pure cannabinoids, not extracts, excluding the entourage effect between cannabis plant components. The vast potential of the phenomenon of synergy between biologically active compounds may be reflected in pharmacotherapy or phytotherapy only after they cross biological barriers, which is only possible for dissolved substances. So far, research to improve the solubility of cannabinoids has focused on improving the solubility of CBD as a result of encapsulation, including nano-emulsions, Pickering emulsions, and inclusion complexes [14]. For example, a recently published article by Wang et al. describes zein and whey protein composite nanoparticles of CBD prepared by a modified anti-solvent method in which the water solubility of CBD was increased by 465–505 times and increased pharmacokinetic parameters [14]. Research to improve the solubility of THC included the use of cyclodextrins in the case of Δ^9^-THC and Δ^8^-THC; for the second substance, it resulted in not only an increase of aqueous solubility but also in the increase of stability and transcorneal permeation [15,16].

Another way to overcome poor cannabinoid solubility in water is by using inhalation as a delivery method in smoking or vaporizing. When a cannabis flower or concentrate is heated to a high enough temperature, the cannabinoids are vaporized and can be inhaled, and they have better bioavailability after inhalation. The value ranges from 10% to 35% for THC and varies among patients due to divergence in number, duration, interval of puffs, breath hold time, inhalation volume, used device, and the site of deposition within the respiratory system; for CBD, the average value is 11–45% [11]. An alternative is to use sublingual drops, which are an extract diluted in a carrier oil to ensure the dissolution of cannabinoids, allowing for rapid absorption through the oral mucosa [17]. The bioavailability of cannabinoids after sublingual administration was assessed for CBD as 13–19%, whilst for THC it was 13–14% [18].

All activities to improve the bioavailability of cannabinoids are aimed at better use of the pharmacological activity of individual cannabinoids—or their mixtures—with a specific potency of individual cannabinoids. Current literature reports confirm neuroprotective, anti-epileptic [19], and sedative effects, which are associated with the achievement of therapeutic goals within the central nervous system. For example, CBD has demonstrated anxiolytic and calming effects in preclinical and clinical studies [20,21,22,23]. By interacting with serotonin receptors and enhancing the action of gamma-aminobutyric acid (GABA), CBD may promote relaxation and potentially aid in managing sleep disturbances and insomnia [24]. The modulation of ion channels, neurotransmitter systems, and anti-inflammatory activity are among the proposed mechanisms through which CBD exerts its antiseizure properties [25]. CBD reduces neuronal excitability through functional antagonism of GPR55 receptors, desensitization of TRPV1 receptors, and inhibition of adenosine transport [26]. Neuroimaging investigations have revealed noteworthy changes in brain activity and connectivity patterns during both resting states and while engaging in cognitive tasks following the administration of CBD [27]. CBD has been found to reduce the accumulation of amyloid-beta (Aβ) plaques and decrease the hyperphosphorylation of tau proteins, which are central pathological features of Alzheimer’s disease [28]. There are not many studies about CBDA or CBC on the nervous system; rather the majority of studies concern CBD and THC. THC interacts with the endocannabinoid system’s CB1 receptors, regulating neurotransmitter release, pain perception, and immune responses [29,30]. However, the use of plant material with a high THC content, even for medicinal purposes, could be deemed illegal in many countries across the globe [31]. The findings indicate that both CBDA and THCA possess properties that may be beneficial in combating Alzheimer’s disease. These cannabinoids can alleviate memory impairments and enhance the brain’s ability to withstand higher levels of calcium (Ca^2+^), Aβ, and hyperphosphorylated tau (p-tau) in the hippocampus [32]. Moreover, a substantial concentration of CBDA effectively reduces neurotoxicity induced by rotenone [32]. In the rat maximal electroshock seizure test, it has been observed that CBDA exhibits anticonvulsant properties [33]. CBC interacts with specific TRP cation channels, namely TRPA1, TRPV1, and TRPV8, which play crucial roles in pain relief and inflammation regulation [34]. Upon binding to these receptors, CBC induces an antinociceptive effect within the brain. CBC positively influenced the viability of adult neural stem progenitor cells during in vitro differentiation, upregulating the marker nestin while downregulating the astrocyte marker Glial fibrillary acidic protein, possibly involving adenosine signaling and ATP modulation in the process [35]. CBC might be also a potential neuronal differentiation inducer for NSC-34 cells (a hybridoma between spinal cord cells from the embryos of mice and neuroblastoma) [36]. In addition to the affinity of cannabinoids to selected receptors, there are also non-specific mechanisms of their action within the central nervous system. There are literature reports, including the results published by us, confirming the scavenging of free radicals [37,38,39]. Recent articles present the variety of antioxidant mechanisms of cannabinoids [38,40,41].

Polyvinyl caprolactam-polyvinyl acetate-polyethylene glycol (Soluplus) is an amphiphilic copolymer composed of hydrophilic and lipophilic segments. This structure allows Soluplus to form micelles or colloidal structures when dispersed in water [42] increasing the solubility of various compounds like curcumin [43], hesperidin [44], pterostilbene [45], or itraconazole [46]. Magnesium aluminometasilicate (Neusilin US2) is an amorphous, porous material with a high surface area and adsorption capacity. Its porous structure can adsorb hydrophobic molecules onto its surface or within its pores and increase the solubility of compounds such as naringenin [47], caffeic acid [48], and celecoxib [49].

**Figure 1:**
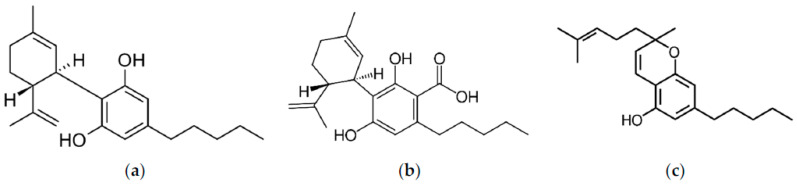
The structure of cannabidiol (**a**), cannabidiolic acid (**b**), and cannabichromene (**c**).

In order to justify the need to increase the solubility and, as a result, the bioavailability of phytocannabinoids present in the inflorescences of *Cannabis* sp., we undertook work to improve the solubility of cannabinoids, CBD, CBDA, and CBC whose structures are presented in Figure 1, by preparing delivery systems with biopolymer (Soluplus) and Neusilin US2 to achieve better bioavailability. Limited research regarding the enhancement of solubility for cannabinoids within whole extracts, rather than isolated or synthesized forms, and notably, the lack of data on dissolution profiles and membrane permeability of CBC and CBDA ensures the novelty of the study.

## 2. Materials and Methods

### 2.1. Materials

Cannabis sativa plant material, Białobrzeskie, Tygra, Henola varieties, was donated from the Experimental Station for the Cultivar Testing in Chrząstowo, belonging to the Research Centre for Cultivar Testing in Słupia Wielka. The agricultural details are presented in Supplementary Materials. The plant material for the study was collected after hemp plants reached the maturation phase, i.e., from the moment of seed formation to the first seed. Immediately after collection, two samples of 500 g each were separated and dried to an absolutely dry mass. The entire drying period lasted twenty hours. The temperature in the oven was maintained at no higher than 50 °C for the first 6 hours and the oven temperature was maintained at 105 °C for the remaining 14 h of drying.

Food-grade CO_2_ was provided by Air Liquide Polska (Cracow, Poland). Soluplus^®^ (polyvinyl caprolactam-polyvinyl acetate-polyethylene glycol graft copolymer), was supplied by BASF SE (Ludwigshafen, Germany). Neusilin US2 (magnesium aluminometasilicate) was kindly provided by Fuji Chemical Industry (Minato, Tokyo). Cannabinoid standards (CBD–CAS: 13956-29-1, CBDA–CAS: 1244-58-2, and CBC–CAS: 20675-51-8) were purchased from Sigma-Aldrich (Poznan, Poland). Trifluoroacetic acid and acetonitrile (high-performance liquid chromatography [HPLC] grade) were provided by Merck (Darmstadt, Germany). The chemicals 2,2-Diphenyl-1-picrylhydrazyl, iron (III) chloride hexahydrate, 2,2′-azino-bis(3-ethylbenzothiazoline-6-sulfonic acid), neocuproine, 2,4,6-Tri(2-pyridyl)-s-triazine, trolox, Trizma^®^ Base, Trizma^®^ hydrochloride, butyrylcholine iodide, acetylcholine iodide, acetylcholinesterase, butyrylcholinesterase, 5,5-dithiobis-2-nitrobenzoic acid, tyrosinase, galantamine, azelaic acid were purchased from Sigma-Aldrich (Schnelldorf, Germany). Sodium chloride, sodium dihydrogen phosphate, sodium hydrogen phosphate, and dimethyl sulfoxide were obtained from Avantor Performance Materials (Gliwice, Poland). Ammonium acetate, an analytical weighed amount of HCl, 1 N, and methanol were supplied by Chempur (Piekary Śląskie, Poland). Cupric chloride dihydrate, acetic acid (99.5%), and ethanol (96%) were supplied by POCH (Gliwice, Poland). Prisma HT, GIT, BBB lipid solution, an acceptor sink buffer, and a brain sink buffer were supplied by Pion Inc. (Forest Row, East Sussex, UK). High-quality pure water was prepared using a Direct-Q 3 UV purification system (Millipore, Molsheim, France; model Exil SA 67120). FaSSIF and FeSSIF were purchased from Biorelevant (London, UK).

### 2.2. Preparation of the Systems of Cannabis sativa (Henola Variety) Inflorescences Extract-Carriers

The extract of *Cannabis sativa* inflorescences was obtained using the dynamic supercritical CO_2_ (scCO_2_) extraction process (SFT-120, shim-pol, Izabelin, Polska). In total, 6.5 g of dried plant material was placed in the extraction vessel and extracted under 6000 psi at 50 °C with 250 mL of CO_2_. The extraction yield was calculated as the mass of extract obtained and subjected to drying (to remove any water from the eventually frozen needle) (g) divided by the mass (g) of plant material placed in the extractor and expressed as a percentage (%). The choice of the Henola extract (HiE) was based on the screening studies on three varieties (Białobrzeskie, Tygra, and Henola) of leaves and inflorescences and their neuroprotective potential (data not presented). After extraction, the antioxidant studies and inhibition of enzymes (acetylcholinesterase, butyrylcholinesterase, and tyrosinase) connected with neurodegeneration were repeated.

**Figure 2:**
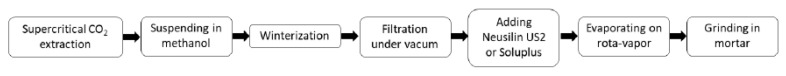
Scheme of preparation of co-dispersion delivery systems of Henola inflorescences extract with Neusilin US2 and Soluplus.

Next, the extracts were dried in a vacuum at 50 °C, weighed, and suspended in methanol (if the process was repeated to obtain more extract, at this stage the extracts were combined together), winterized, and filtered (Figure 2). For fluid extracts (HiE), carriers (Neusilin US2, Soluplus, or lactose for apparent solubility study) were added in a 1:1 mass ratio to the earlier weight of the extract. Systems were dried on rota-vapor at 50 °C until dry and grounded in mortar.

### 2.3. Chromatographic Analysis

The cannabinoid profile (CBD, CBDA, and CBC) of the extract, and during the apparent solubility and permeability study, was analyzed using the ultra-high-performance liquid chromatography with the diode array detector (HPLC-DAD) method, Shimadzu Corp. (Kyoto, Japan). The previously described method was used [37]. The analysis was conducted on a CORTECS Shield RP18 stationary phase, 2.7 µm; 150 mm × 4.6 mm, with a mobile phase consisting of 0.1% trifluoroacetic acid (41%) and acetonitrile (41:59, *v*/*v*). The flow rate was set to 2.0 mL/min, and the column temperature was maintained at 35 °C. The injection volume was 10.0 µL, and the detection wavelength was set at 228 nm, with an analysis time of 50 min. The retention times for each cannabinoid were as follows: CBD at approximately 5.83 min, CBDA at approximately 6.42 min, and CBC at 14.57 min. The LabSolutions LC software (version 1.86 SP2) from Shimadzu Corp. (Kyoto, Japan) was used to obtain chromatograms. The method was validated according to ICH guidelines for current research, the validation parameters are collected in Table S1 (Supplementary Materials).

### 2.4. Apparent Solubility of Cannabinoids

The dissolution rate was determined in the paddle apparatus (Agilent Technologies, Santa Clara, CA, USA). HiE had a thick, oily consistency, so for the purpose of apparent solubility study it was combined with lactose; the preparation steps were the same as for Neusilin US2 and Soluplus (HiE–control). The systems and control (600 mg) were placed into two gelatin capsules. The capsules were placed into coiled sinkers for floating prevention. The test was carried out in triplicate for 180 min in a pH 1.2 of 0.1 N hydrochloric acid, a pH 6.8 of phosphate buffer, Fasted State Simulated Intestinal Fluid (FaSSIF), and Fed State Simulated Intestinal Fluid (FeSSIF).

FaSSIF and FeSSIF dissolution media are more complex solutions specifically designed to simulate the conditions of the human small intestine under fasted and fed conditions. FaSSIF and FeSSIF contain natural surfactants present in the gut to simulate gastrointestinal fluids much more accurately than conventional dissolution media, and they simulate the conditions of the human intestine in a fasted state and after a meal [50]. Sodium taurocholate is included to replicate the role of bile acids in facilitating lipid absorption and emulsification. Lecithin is incorporated to mimic the presence of phospholipids, which play a vital role in the formation of mixed micelles that enhance the solubilization of lipophilic compounds. The buffer ensures a stable pH in the intestinal fluid (6.5 for FaSSIF and 5.0 for FeSSIF), and sodium chloride is added to ensure physiological osmolarity (a FaSSIF of 270 Osm/L and FeSSIF of 670 Osm/L) [51].

The vessels were filled with 500 mL of media at the temperature set at 310.15 K and the rotation speed of 100 rpm. At specific time intervals, 2.0 mL of the sample was taken out and immediately replaced with an equal amount of fresh medium at the same temperature. The percentage cumulative cannabinoid release (% CBD, CBDA, and CBC) was measured at different time points (5, 10, 15, 30, 45, 60, 90, 120, and 180 min) for each formulation. The samples were then passed through a filter with a pore size of 0.22 μm and analyzed using high-performance liquid chromatography (HPLC). Sample chromatograms from the dissolution study are presented in Figure S1 (Supplementary Materials). The standard deviation (SD) was also calculated for each time point and delivery system.

The differences and similarities between the apparent solubility profiles were determined by the two-factor values, *f*_1_ and *f*_2_, introduced by Moore and Flanner [52] with the use of the following equations:

(1) $$f_{1}=\frac{\sum_{j=1}^{n}\left|R_{j}-T_{j}\right|}{\sum_{j=1}^{n}R_{j}}$$

(2) $$f_{2}=50\times \log\left({\left(1+\left(\frac{1}{n}\right)\sum_{j=1}^{n}{\left|R_{j}-T_{j}\right|}^{2}\right)}^{-\frac{1}{2}}\times 100\right)$$

where *n* is the number of time points, *R_j_* is the percentage of the reference dissolved substance in the medium, *T_j_* is the percentage of the dissolved tested substance, and t is the time point. Dissolution profiles are described as similar when the *f*_1_ value is close to 0, or *f*_2_ is close to 100 (between 50 and 100) [53]. The similarities and dissimilarities between profiles were marked in the figures with letters. If profiles share the same letter, they are similar.

The data from the dissolution studies were graphically correlated to mathematical models: zero-order, first-order, Higuchi’s model, and Korsmeyer–Peppas model in MS Excel (version 1808, Microsoft Corporation, Redmond, WA, USA) [54,55]. The mathematical equations of kinetic models are described below:

Zero-order model: F = k × t

First-order model: lnF = k × t

Higuchi model: F = kt^1/2^

Korsmeyer–Peppas model: F = kt^n^

where F is the fraction of the released drug, k is the constant associated with the release, and t is the time (h).

### 2.5. Permeability Study of Cannabinoids

The permeability of cannabinoids through biological membranes was measured using the Parallel Artificial Membrane Permeability Assay (PAMPA) model. The study was conducted in the gastrointestinal (GIT) and blood-brain barrier (BBB) models. The model consists of two 96-well microfilter plates, the donor and the acceptor plate. The wells were separated by a 120 μm thick microfilter disc coated with a 20% (*w*/*v*) dodecane solution of a lecithin mixture (Pion Inc., Billerica, MA, USA). The extract was diluted and the systems were dissolved (or suspended, centrifuged, and filtered) in dimethyl sulfoxide (DMSO) and placed in the donor solutions, which were adjusted to 6.8 for GIT application and pH 7.4 for BBB. The BBB permeability was only studied for extract, as Neusilin US2 does not leave the GIT. The plates were incubated at 310.15 K for 3 h for the GIT and BBB assay in a humidity-saturated atmosphere. After incubation, the plates were separated and the concentration of CBD and CBDA, as their concentration in the extract was the highest, was determined using the HPLC-DAD method. Each measurement was repeated six times. CBC was present in a quantifiable concentration only in the BBB study, thus, it was not determined in GIT conditions. The *P_app_* was calculated using the following formulas:

(3) $$P_{app}=\frac{-\ln\left(1-\frac{C_{A}}{C_{equilibrium}}\right)}{S\times \left(\frac{1}{V_{D}}+\frac{1}{V_{A}}\right)\times t}$$

(4) $$C_{equilibrium}=\frac{C_{D}\times V_{D}+C_{A}\times V_{A}}{V_{D}+V_{A}}$$

where *V_D_* is the donor volume, *V_A_* is the acceptor volume, *C_equilibrium_* is the equilibrium concentration ($C_{equilibrium}=\frac{C_{D}\times V_{D}+C_{A}\times V_{A}}{V_{D}+V_{A}}$), *S* is the membrane area, and *t* is the incubation time (in seconds).

Substances with a *P_app_* in the GIT model below 0.1 × 10^−6^ cm/s are considered to have poor permeability, compounds with 0.1 × 10^−6^ cm/s ≤ *P_app_* < 1 × 10^−6^ cm/s are classified as mediocre permeable, and compounds found as well permeable have a *P_app_* ≥ 1 × 10^−6^ cm/s [56]. Compounds whose *P_app_* in the BBB model is <2.0 × 10^−6^ cm/s are known as poorly permeable. Compounds with questionable permeability have *P_app_* values in the range of 2.0 to 4.0 × 10^−6^ cm/s. Substances that have a *P_app_* value greater than 4.0 × 10^−6^ cm/s are regarded as highly permeable [57].

### 2.6. Biological Activity Studies

The extract and systems antioxidant activity was studied by four assays: DPPH, ABTS, CUPRAC, and FRAP. Two of them determine the ability to scavenge free radicals (DPPH and ABTS), whilst the other assays check the possibility of performing redox reactions (CUPRAC and FRAP). A linear regression equation between the trolox concentration and its scavenging percentage (DPPH and ABTS) or absorbance (CUPRAC and FRAP) was built. Thus, the results, presented as mg trolox/g plant material, were calculated through the equation according to the antioxidant properties of the extracts in all four assays [58,59]. Pure excipients showed no antioxidant potential under test conditions.

To perform the DPPH assay, a 96-well plate was used and the samples were measured spectrophotometrically [60]. The main reagent was a methanol solution of DPPH at a concentration of 0.2 mM. To initiate the assay, 25.0 µL of the system/trolox solution was mixed with 175.0 µL of the DPPH solution. The plate was then incubated in the dark at room temperature while shaking for 30 min. After the incubation period, the absorbances were obtained using a plate reader (Multiskan GO, Thermo Fisher Scientific, Waltham, MA, USA) at 517 nm. The absorbance (A) was also measured for a blank sample, which consisted of a mixture of DPPH solution and solvent at 517 nm. Each sample was tested for its absorbance at 517 nm. The inhibition of DPPH radicals by the studies’ systems/trolox was calculated using the equation:

(5) $$DPPH\;scavenging\;activity\;(\%)\;=\;\frac{A_{o\;}-A_{i}}{A_{o}}\times 100\%$$

where *A_o_* is the absorbance of the control sample and *A_i_* is the absorbance of the test sample. Each measurement was repeated six times.

As another assay to determine the scavenging radical potential, the ABTS study [61], was also performed. This study is based on the production of green cation radicals through the loss of electrons by nitrogen atoms of ABTS caused by potassium persulfate. During the assay, the green ABTS radical can be converted into a colorless neutral form in the presence of an antioxidant. In this assay, 200.0 μL of ABTS^•+^ solution and 10.0 μL of the system/trolox solution were pipetted into 96-well plates and incubated for 10 min in the dark at room temperature while shaking [62]. After incubation, the absorbance values were measured at λ = 734 nm using a plate reader (Multiskan GO, Thermo Fisher Scientific, Waltham, MA, USA). The mixture of solvent and ABTS (control) and the wells filled with system and water (systems’ absorbance) at 734 nm were also studied. The inhibition of ABTS^•+^ was calculated using the following equation:

(6) $$\mathrm{ABTS}\;\mathrm{scavenging}\;\mathrm{activity}\;(\%)=\frac{A_{0}-A_{1}}{A_{0}}\times 100\%$$

where:

*A*_0_—The absorbance of the control;

*A*_1_—The absorbance of the sample.

To determine the reducing potential of the systems, the CUPRAC assay [63] was used. In this assay, the antioxidants’ phenolic groups undergo oxidation to form quinones, while the bluish neocuproine and copper (II) ion complex is reduced to the yellow neocuproine and copper (I) ion complex. To perform this study, a mixture of 50.0 µL of the system/trolox solution and 150.0 µL of the CUPRAC reagent was added to the plate and then incubated for 30 min at room temperature while shaking in the dark [62]. The control and systems’ own absorbance were also measured simultaneously. The absorbance was measured at a wavelength of 450 nm using a plate reader (Multiskan GO, Thermo Fisher Scientific, Waltham, MA, USA) after the 30 min incubation period. The analysis was performed using six replicates.

The FRAP technique was also used to determine the reducing properties of the systems, which involves reducing colorless Fe^3+^ ion to Fe^2+^ to form a dark blue complex with 2,4,6-tris(2-pyridyl)-1,3,5-triazine (TPTZ) [62]. In this method, 25.0 µL of the system/trolox solution and 175.0 µL of the FRAP mixture (consisting of 25 mL acetate buffer, 2.5 mL TPTZ solution, and 2.5 mL of FeCl_3_·6H_2_O solution) were applied to the plate and incubated in dark conditions at 37 °C for 30 min. The control and systems’ absorbance were also measured. Subsequently, the absorbance was measured at λ = 593 nm using a plate reader (Multiskan GO, Thermo Fisher Scientific, Waltham, MA, USA). The analysis was performed using six replicates.

The neuroprotective effect of cannabinoids was assessed against the possibility of inhibiting enzymes whose expression is associated with neurodegenerative changes.

As a standard inhibitor of esterases, galantamine was chosen, while for tyrosinase, azelaic acid was selected [64,65]. A linear regression equation that relates the standard concentration of a substance to its ability to inhibit an enzyme, as measured by the percentage of potential inhibition was created. An equation to calculate the standard equivalent for each extract based on its inhibitory properties in all three assays was obtained. The results were presented as a galantamine equivalent (GALAE) (mg galantamine/g plant material) for AChe and BChE assays and as an azelaic acid equivalent (AzAE) (mg azelaic acid/g plant material) [66,67,68,69,70,71].

The inhibition of acetylcholinesterase (AChE) and butyrylcholinesterase (BChE) was carried out using a colorimetric Ellman et al. modified assay [72]. This method requires artificial substrates (thiocholine esters). Thiocholine is liberated during the enzymatic reactions with 5,5′-dithio-bis-(2-nitrobenzoic) acid (DTNB), and the 3-carboxy-4-nitrothiolate anion (TNB anion) is formed. The potential to inhibit AChE and BChe was measured according to the increase in the thiocholine color in a 96-well plate. In total, 60.0 μL of 0.05 M Tris-HCl buffer (pH of 8.0), 10.0 μL of test solution, and 30.0 μL of AChE/BChE solution at a concentration of 0.2 U/mL were added to the wells. Subsequently, the plate was incubated for 5 min at 37 °C while shaking. Next, 30.0 μL acetylthiocholine iodide (ATCI)/butyrylthiocholine iodide (BTCI) at a concentration of 1.5 mM and 125.0 μL of 0.3 mM DTNB solution (5,5′-dithiobis-(2-nitrobenzoic acid)) were added to the wells. The plate was then incubated for another 20 min under the same conditions. A blank sample (the reaction mixture without the enzyme, with an increase in the volume of Tris-HCl buffer), a control sample (the solvent instead of the test sample), and a blank sample for the control sample (the reaction mixture without the enzyme, with an increase in the volume of Tris-HCl buffer) were also prepared. The measurements were performed at a wavelength of 405 nm. The analysis was performed using six replicates. The percentage of inhibition of AChE and BChE by the test samples was calculated using the following formula:

(7) $$\mathrm{AChE}/\mathrm{BChE}\;\mathrm{inhibition}\;(\%)=\frac{1-(A_{1}-A_{1b})}{\left(A_{0}-A_{0b}\right)}\times 100\%$$

where:

*A*_1_—The absorbance of the test sample;

*A*_1*b*_—The absorbance of the blank of the test sample;

*A*_0_—The absorbance of control;

*A*_0*b*_—The absorbance of the blank of control.

The tyrosinase inhibition assay measures the activity of an inhibitor to prevent L-DOPA from accessing the tyrosinase active site. This leads to a decrease in the color intensity of the solution, which indicates enzyme inhibition [73]. To conduct the assay, 75.0 μL of 0.1 M phosphate buffer (pH 6.8) was added to each well of a 96-well plate, followed by 25.0 μL of the extract and 50.0 μL of enzyme solution (192 U/mL). The plate was shaken at room temperature for 10 min, after which 50 μL of 2.0 mM L-DOPA was added to each well and incubated for an additional 20 min under the same conditions. In addition to the test sample, a blank for the test sample (without enzyme, the volume of phosphate buffer was elevated), a control sample (with solvent instead of the test sample), and a blank sample for the control (without enzyme) were also prepared. The absorbance of the samples was measured at 475 nm. Each measurement was repeated six times. The percentage inhibition of the tyrosinase by the samples was calculated using an equation:

(8) $$\mathrm{Tyrosinase}\;\mathrm{inhibition}\;(\%)=\frac{1-(A_{1}-A_{1b})}{\left(A_{0}-A_{0b}\right)}\times 100\%$$

where:

*A*_1_—The absorbance of the test sample;

*A*_1*b*_—The absorbance of the blank of the test sample;

*A*_0_—The absorbance of control;

*A*_0*b*_—The absorbance of the blank of control.

### 2.7. Statistical Analysis

Statistical analysis of results obtained in permeability assay, and in antioxidant activity study, was performed with the use of Statistica 13.3 software (StatSoft Poland, Krakow, Poland). Data are presented as mean values ± standard deviations. Experimental data were analyzed using the skewness and kurtosis tests to determine the normality of each distribution, and Levene’s test assessed the equality of variances. Statistical significance was determined using a one-way analysis of variance (ANOVA), followed by the Bonferroni post hoc test (to compare the experimental results acquired for cannabinoids in extract and in the systems). Differences were considered significant at *p* < 0.05.

## 3. Results

### 3.1. Preparation and Characterization of Co-Dispersion Delivery Systems

Using extracts obtained from inflorescences with the scCO_2_ extraction technique (the extraction yield was ~16.74%), cannabinoid delivery systems with increased solubility and permeability were obtained. As model carriers, biopolymer Soluplus and Neusilin US2 were applied. The systems of cannabinoids with carriers (Figure 2) were prepared using a solvent-evaporation method which enables the incorporation of a wide range of active ingredients into the resulting systems [74,75].

The extracts and systems have undergone the HPLC-DAD analysis to determine the cannabinoid content. In HiE, CBD was at the level of 6042.76 ± 82.19 μg/g plant material, CBDA at 2033.01 ± 67.98 μg/g plant material, whilst for CBC, 238.71 ± 11.20 μg/g plant material. The results of the systems analysis are presented in Table 1.

### 3.2. Apparent Solubility of Cannabinoids

Two systems of the HiE with Neusilin US2, and Sol prepared in a 1:1 mass ratio (extract weight: carrier) using a solvent-evaporation technique, were enrolled in the dissolution study. The percentage cumulative cannabinoid release (% CBD, CBDA, and CBC) was measured at different time points (5, 10, 15, 30, 45, 60, 90, 120, and 180 min) for each system.

**Figure 3:**
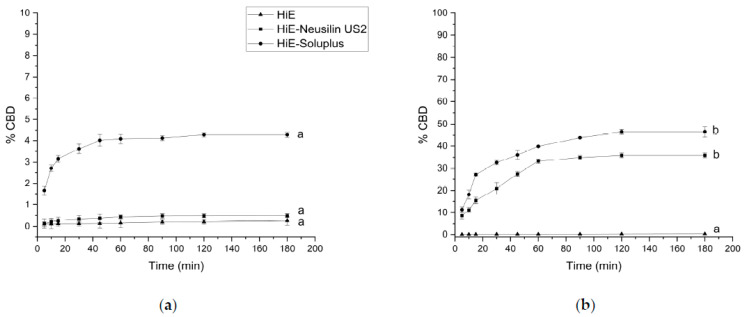
The dissolution profiles of CBD from HiE, HiE-Neusilin US2, and HiE-Soluplus systems at pH 1.2 (**a**) and 6.8 (**b**). Profiles with the same superscript letters were similar (according to *f*_1_ or *f*_2_ values). Profiles with different superscript letters differ significantly (according to *f*_1_ and f_2_ values).

In 0.1 M hydrochloric acid, at pH 1.2 (Figure 3a), CBD was dissolved to the smallest extent compared to other media. After 60 min of the study, the percentage of dissolved CBD is only in HiE-Soluplus 4.08% ± 0.21%, in HiE-Neusilin US2 0.44% ± 0.09%, and even less in HiE. In the phosphate buffer at pH 6.8 (Figure 3b), the dissolution rate of CBD was overall greater for CBD in the co-dispersion delivery systems than in pH 1.2; however, CBD from HiE did not dissolve. After 60 min, the % CBD values were as follows: HiE-Soluplus at 39.83% ± 0.23% and HiE-Neusilin US2 at 33.21% ± 1.09%. CBD had the highest dissolution rate in HiE-Soluplus at pH 6.8 throughout the whole study.

**Figure 4:**
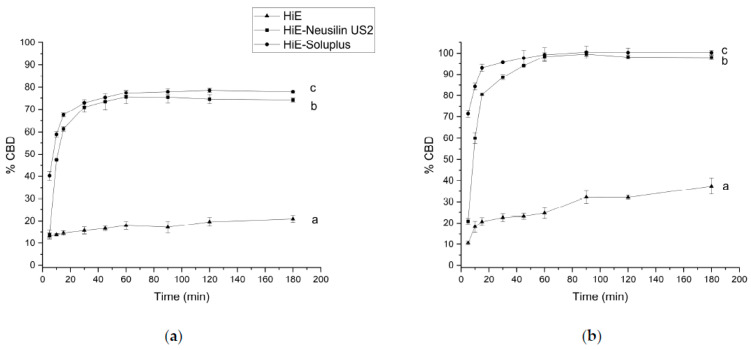
The dissolution profiles of CBD from HiE, HiE-Neusilin US2, and HiE-Soluplus systems in FaSSIF (**a**) and FeSSIF (**b**). Profiles with different superscript letters (a–c) differ significantly (according to *f*_1_ and *f*_2_ values).

The apparent solubility of CBD was also studied in FaSSIF and FeSSIF (Figure 4a,b). The dissolution profile of CBD was greater in FaSSIF and FeSSIF than in pharmacopeial media at pH 1.2 and 6.8. It is observed that in both advanced media, CBD was rapidly released from co-dispersion delivery systems. After 60 min, in FaSSIF, CBD was dissolved in HiE-Soluplus at 77.40% ± 1.15%, in HiE-Neusilin US2 at 75.47% ± 2.91%, and in HiE at 17.92% ± 1.79%. In FeSSIF (Figure 4b), CBD was released to the greatest extent, reaching after 60 min in HiE-Soluplus 99.25% ± 3.23%, in HiE-Neusilin US2 98.37% ± 1.82%, and in HiE 24.76% ± 2.48%. Co-dispersion delivery system HiE-Soluplus provided the best dissolution rate of CBD at each time point, which was statistically significantly different than CBD dissolution profiles in HiE-Neusilin US2 and HiE.

The dissolution of CBD is a complex process influenced by various factors. The dissolution kinetics of CBD were investigated using various mathematical models under different media conditions and in extract and co-dispersion delivery systems with Neusilin US2 and Soluplus (Table 2) [76,77,78,79]. Four mathematical models, namely zero-order kinetics, first-order kinetics, Higuchi kinetics, and Korsmeyer–Peppas kinetics were employed to analyze the dissolution data [80,81,82]. CBD in HiE displayed high R^2^ values for zero-order and first-order kinetics, indicating a reliable and predictable release mechanism. The Higuchi model also showed notable correlations, suggesting diffusion-driven release. Moreover, the Korsmeyer–Peppas model displayed moderate to high correlations, and the n values indicated a Fickian diffusion. For both HiE-Soluplus and HiE-Neusilin US2, the Higuchi model consistently revealed diffusion-driven release mechanisms across pH conditions and biorelevant media. The Korsmeyer–Peppas model, which was also dominating for CBD in co-dispersion delivery systems, indicated the involvement of Fickian transport based on the n values (n < 0.5) [83].

**Figure 5:**
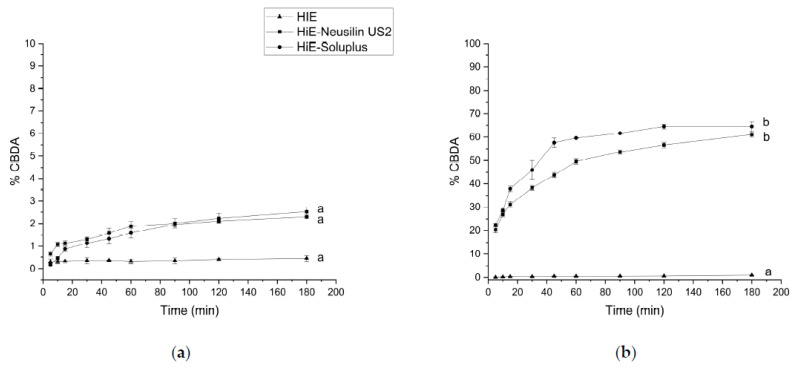
The dissolution profiles of CBDA from HiE, HiE-Neusilin US2, and HiE-Soluplus systems at pH 1.2 (**a**) and 6.8 (**b**). Profiles with the same superscript letters were similar (according to *f*_1_ or *f*_2_ value). Profiles with different superscript letters differ significantly (according to *f*_1_ and *f*_2_ values).

The CBDA dissolution rate was also monitored under the same conditions. In hydrochloric acid, at pH 1.2, the overall results were the poorest (Figure 5a), it practically did not dissolve from HiE. After 60 min of the assay, CBDA was dissolved in HiE-Soluplus and HiE-Neusilin US2 at the level of 1.58% ± 0.21% and 1.88% ± 0.21%, respectively. The co-dispersion delivery system HiE-Soluplus provided the greatest dissolution rate of CBDA at pH 1.2. However, the overall results are poor and the profiles are statistically similar. The apparent solubility of CBDA was also studied in a phosphate buffer at pH 6.8 (Figure 5b). Similarly, to pH 1.2, CBDA in HiE practically did not dissolve during the study. After 60 min, HiE-Soluplus had a CBDA dissolution rate of 59.56% ± 0.23%, while HiE-Neusilin US2 was 49.64% ± 1.09%.

**Figure 6:**
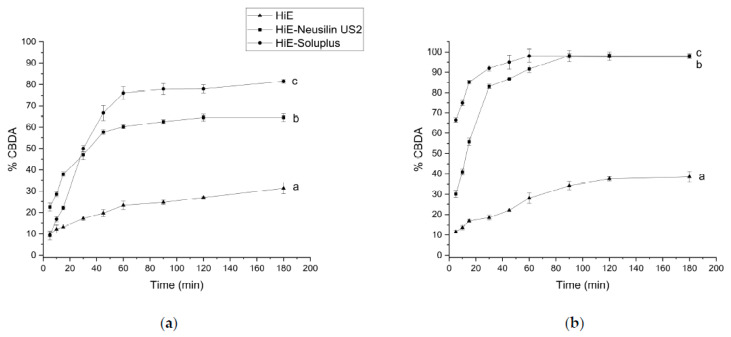
The dissolution profiles of CBDA from HiE, HiE-Neusilin US2, and HiE-Soluplus systems in FaSSIF (**a**) and FeSSIF (**b**). Profiles with different superscript letters (a–c) differ significantly (according to *f*_1_ and *f*_2_ values).

As for CBD, CBDA was also studied in FaSSIF (Figure 6a). The most noticeable differences are noted at the beginning of the study. After one hour of the assay, the dissolution percentages for CBDA in HiE-Soluplus, HiE-Neusilin US2, and HiE were 76.05% ± 2.91%, 60.20% ± 0.96%, and 23.25% ± 1.87% respectively. The results indicate that CBDA was dissolved to the greatest extent in HiE-Soluplus, which was statistically better than in HiE-Neusilin US2 and HiE. In the FeSSIF medium, the CBDA dissolution profile in HiE-Soluplus reaches the highest dissolution rate values and it differs significantly from the CBDA profile in HiE-Neusilin US2 and HiE (Figure 6b). The first time point, 5 min, shows the biggest variability in CBDA dissolution rate, where the percentage of CBDA released was 66.56% ± 1.09% for HiE-Soluplus, 30.13% ± 1.68% for HiE-Neusilin US2, and 11.52% ± 0.65% for HiE. The maximum dissolution rates are higher in FeSSIF than in FaSSIF.

The dissolution kinetics of CBDA was also studied (Table 3). CBDA consistently displayed the highest R^2^ values in the Korsmeyer–Peppas and Higuchi models. The n values, fluctuating mostly from below 0.45 to three values below 0.89, suggest a potential dominance of Fickian diffusion. In three cases, the n values between 0.45 and 0.89 indicated the non-Fickian diffusion release mechanism which shows the relative complexity of the prepared co-dispersion delivery systems and may indicate that the CBDA release is controlled by more than one mechanism.

**Figure 7:**
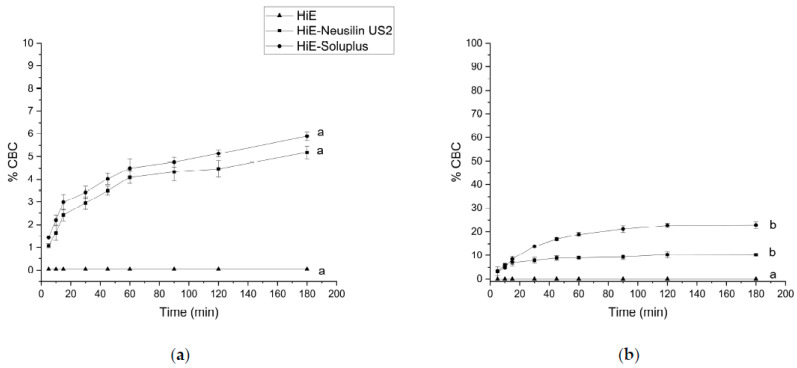
The dissolution profiles of CBC from HiE-Neusilin US2 and HiE-Soluplus systems at pH 1.2 (**a**) and 6.8 (**b**). Profiles with the same superscript letters were similar (according to *f*_1_ or *f*_2_ value). Profiles with different superscript letters differ significantly (according to *f*_1_ and *f*_2_ values).

Following the methodology used for the apparent solubility study of CBD and CBDA, the dissolution profiles for CBC were determined in the same media and time points. In the study conducted at pH 1.2 (Figure 7a), the dissolution rate was similar for CBD and CBDA (the lowest). CBC did not dissolve in HiE during the study. At the last time point, 180 min, HiE-Soluplus showed the highest percentage of CBC released at 5.89%, whilst in HiE-Neusilin US2, CBC was dissolved in 5.18% ± 0.28%. CBC profiles were similar due to *f*_1_ and *f*_2_ factors. In the study where vessels were filled with phosphate buffer at pH 6.8 (Figure 7b), CBC in HiE was not dissolved, and the most noticeable differences were noted in the first minutes of the study. The CBC reached in HiE-Soluplus (120 min) of the study was 10.30% ± 1.36%. Whilst in HiE-Neusilin US2, it was 22.75% ± 1.00%. Both dissolution profiles of CBC in co-dispersion delivery systems were similar.

**Figure 8:**
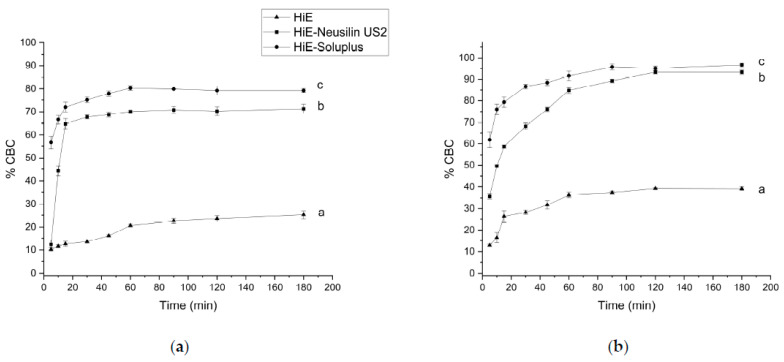
The dissolution profiles of CBC from HiE-Neusilin US2 and HiE-Soluplus systems in FaSSIF (**a**) and FeSSIF (**b**). Profiles with the same superscript letters were similar (according to *f*_1_ or *f*_2_ value). Profiles with different superscript letters (a–c) differ significantly (according to *f*_1_ and *f*_2_ values).

In fasted state intestinal conditions, the most dynamic changes, take place at 5 min of the study, where CBC is dissolved in HiE-Soluplus at 56.63% ± 2.80%, in HiE-Neusilin US2 at 12.28% ± 2.11%, and in HiE at 10.26% ± 0.81% (Figure 8a). After 30 min, CBC reached a plateau. The CBC profile in HiE-Soluplus is significantly better than in HiE-Neusilin US2 and HiE. The last environment in which the CBC dissolution rate was studied was FeSSIF (Figure 8b), where the greatest dissolution rate of CBC was obtained. After 15 min of the study, CBC was dissolved in 79.32% ± 2.30%, 58.68% ± 0.57%, and 26.25% ± 2.79% in HiE-Soluplus, HiE-Neusilin US2, and HiE, respectively. The CBC dissolution profile in HiE-Soluplus was significantly better than in HiE-Neusilin US2 and HiE.

The Higuchi and Korsmeyer–Peppas models consistently yield higher R^2^ values compared to the zero-order and first-order models across different CBC formulations and pH conditions (Table 4). The release exponent (n) values are consistently below 0.5 across formulations and pH conditions, suggesting the release approximated the Fickian diffusion release mechanism indicative of controlled release predominantly driven by diffusion.

The results showed that HiE-Soluplus consistently provided the highest dissolution rate of cannabinoids compared to HiE-Neusilin US2 and HiE. The dissolution rate of CBD, CBDA, and CBC was highest in FeSSIF, followed by FaSSIF and the phosphate buffer at pH 6.8, while the lowest dissolution rate was observed in 0.1 M hydrochloric acid at pH 1.2. At pH 1.2, all three cannabinoids showed poor solubility. In a phosphate buffer with a pH of 6.8, the greatest improvement in solubility was observed for CBDA. CBDA, being the acidic precursor of CBD, might have some advantages in solubility compared to CBD and CBC. In FaSSIF, the maximum dissolution rate was similar for CBD, CBDA, and CBC. However, the fastest increase in dissolution rate was noted for CBC. In FeSSIF, the dissolution profiles for CBD, CBDA, and CBC were again similar, but the fastest increase in dissolution rate was observed for CBD. FaSSIF and FeSSIF provided a more similar composition to intestinal fluid than the pharmacopoeial media, containing surfactants that helped significantly increase the solubility of cannabinoids.

### 3.3. Permeability Study

Increasing gastrointestinal permeability is important to obtain higher bioavailability as it allows for more efficient absorption into the bloodstream from the GI tract. Thus, a PAMPA study was performed.

The permeability coefficients of CBD in pH 6.8 were analyzed in HiE and co-dispersion delivery systems: HiE-Neusilin US2 and HiE-Soluplus (Table 5). The highest permeability coefficient was observed in HiE-Soluplus (3.09 × 10^−7^ ± 1.07 × 10^−8^ cm/s), followed by HiE-Neusilin US2 (2.73 × 10^−7^ ± 9.75 × 10^−9^ cm/s), and the CBD permeability was statistically the worst in the pure extract (1.86 × 10^−7^ ± 2.24 × 10^−8^ cm/s).

CBDA was better permeable than CBD through membranes in the study under the same conditions (Table 5). Its permeability coefficient reached 7.57 × 10^−6^ ± 1.21 × 10^−7^ cm/s in HiE, while the most noticeable and statistically significant increase was found in HiE-Soluplus, where CBDA reached 9.51 × 10^−6^ ± 4.66 × 10^−8^ cm/s and 7.56 × 10^−6^ ± 2.69 × 10^−7^ cm/s in HiE-Neusilin US2.

The BBB permeability was assessed for CBD, CBDA, and CBC in HiE. All P_app_ values were determined as higher than 4.0 × 10^−6^ cm/s, meaning that both cannabinoids cross the blood-brain barrier well.

### 3.4. Biological Activity Studies

HiE extract and the systems showed antioxidant activity (Table 6). In the DPPH model, the best result was obtained for Hi-Soluplus (0.97 ± 0.02 mg trolox/g plant material), while for HiE (0.85 ± 0.01 mg trolox/g plant material), however, these results are statistically similar. In the other scavenging radicals assay, ABTS, the HiE (17.04 ± 0.08 mg trolox/g plant material) antioxidant potential was improved the most by HiE-Soluplus (18.69 ± 0.17 mg trolox/g plant material). In the CUPRAC redox study, the greatest result was obtained for HiE-Soluplus (7.81 ± 0.22 mg trolox/g plant material). In FRAP, the most noticeable improvement in HiE antioxidant activity (11.58 ± 0.03 mg trolox/g plant material) was shown also for HiE-Soluplus (1.65 ± 0.03 mg trolox/g plant material). In general, the results show statistically significant improvement in ABTS and FRAP assays in antioxidant potential in the systems when compared to HiE, but the changes are subtle.

The inhibition of the enzymes connected to the development of neurodegeneration was also studied for the extract (Table 7). HiE inhibited an AChE of 20.78 ± 0.56 mg galantamine/g, while BChE was 17.49 ± 0.47 mg galantamine/g. Tyrosinase was also inhibited by the HiE 165.21 ± 7.11 mg azelaic acid/g. Preparation of the systems increased the inhibitory activity of the cannabinoids. The greatest enhancement was noted for HiE-Soluplus (AChE 21.06 ± 0.19 mg galantamine/g, BChE 17.54 ± 0.09 mg galantamine/g, and tyrosinase 171.30 ± 2.13 mg azelaic acid/g). The changes in biological activity are subtle, but there is a visible trend that the neuroprotective potential is increasing.

## 4. Discussion

Henola inflorescences were extracted with scCO_2_. scCO_2_ extraction is widely recognized as a green extraction method. CO_2_ functions as a non-polar solvent, and in its supercritical state, it is a good choice for efficiently extracting lipophilic compounds like cannabinoids from plant material [84]. Its selectivity, safety, and environmentally friendly characteristics further contribute to its suitability for this purpose [85,86]. Furthermore, the use of CO_2_ as a solvent eliminates the need for harsh organic solvents, resulting in a pure extract without the risk of residual solvent contamination [87,88,89]. scCO_2_ extraction is particularly advantageous for extracting cannabinoids from cannabis plant material due to the lipophilic character of these compounds. In its supercritical state, CO_2_ exhibits both gas-like diffusion and liquid-like solvency, allowing it to penetrate the plant matrix efficiently and dissolve target compounds [90]. Alcohol extraction is a common method for cannabinoids, extracting a wide range of compounds, but it may also pull undesirable components like chlorophyll and is considered a less environmentally friendly method than scCO_2_ [91]. Hydrocarbon extraction offers high yields due to its strong solvent power, yet it poses safety risks due to flammable solvents and requires extensive post-extraction purification [91]. Unlike solvent-based techniques, such as ethanol or hydrocarbon extraction, scCO_2_ is a non-toxic solvent that leaves no residual solvents in the final product, ensuring the purity and safety of extracted cannabinoids. Co-dispersion delivery systems of HiE with Neusilin US2 and Soluplus were prepared with a solvent-evaporation technique, which is a relatively simple, cost-effective, and scalable method [92]. The straightforward nature of the method allows for efficient and cost-effective production of bulk quantities of the desired systems, making it suitable for various industrial applications including pharmaceuticals, cosmetics, and materials engineering. An important aspect was obtaining co-dispersions in powder form for a future oral formulation, which was provided by both Neusilin US2 and Soluplus.

CBD, CBDA, and CBC from HiE did not dissolve in the pharmacopeial media (pH 1.2 and 6.8) as 1% of dissolution was not exceeded in any case within 180 min of the study. Considering CBDA’s approximate pKa value of 2.9 [93], CBDA primarily exists in its acidic form under both acidic (pH 1.2) and neutral (pH 6.8) conditions. The improved dissolution rate at pH 6.8 suggests that the neutral environment favors CBDA solubility and release. The approximate pKa value of CBC is around 9.5–10.3 [94], and it is similar to CBD (pKa 9.3–10.3 [94,95]). In the highly acidic environment of pH 1.2, CBC exhibited a similarly low dissolution rate compared to CBD and CBDA. These results suggest that CBC, like CBD and CBDA, has limited solubility and dissolution in highly acidic conditions, which may be attributed to its weakly basic nature. In a phosphate buffer at pH 6.8, CBC demonstrated improved dissolution rates compared to pH 1.2, suggesting enhanced solubility in more neutral environments. In view of the literature reports, the decomposition of cannabinoids in an acidic environment during this study cannot be ruled out [96]. It can, therefore, be assumed that such a low percentage of cannabinoid release in acidic conditions might be due to the poor solubility in the stomach environment, but also due to the degradation of the CBD, CBDA, or CBC.

The solubility and dissolution behavior of cannabinoids, such as CBD, CBDA, and CBC, can vary significantly depending on the pH of the surrounding environment and the presence of specific surfactants. This is visible in the case of the dissolution of cannabinoids from HiE before co-dispersion delivery systems preparation in FaSSIF and FeSSIF as they reached 20–31% and 37–40%, respectively. The dissolution profile of cannabinoids was notably higher in both FaSSIF and FeSSIF compared to the pharmacopeial media. This indicates that the presence of natural surfactants present in FaSSIF and FeSSIF better simulates the complex environment of the gut, leading to a more efficient release of CBD, CBDA, and CBC. Surfactants are amphiphilic molecules, meaning they have both hydrophobic and hydrophilic regions. The presence of surfactants in the solution can help solubilize cannabinoids by forming micelles or emulsions. What is more important, the better solubility of cannabinoids in post-meal conditions was indicated, which was also proven in other studies [97]. The increase in the bioaccessibility of CBD with food could be explained by the fact that micelle formation from hydrolyzed lipids aids in the bioaccessibility of hydrophobic molecules [98]. How cannabinoids are administered, as well as the meal with which they are taken, is a very important aspect to receive the appropriate pharmacological response.

An increase in apparent solubility of cannabinoids was obtained due to co-dispersion delivery systems with Neusilin US2 and Soluplus. Neusilin US2 is a type of synthetic magnesium aluminosilicate which is a porous material with a significant surface area, porosity, and adsorption capacity. Its structure consists of a three-dimensional network of interconnected particles with numerous pores and channels. When Neusilin US2 is in contact with water, its porous structure can adsorb hydrophobic molecules like cannabinoids onto its surface or within its pores [99]. This adsorption effectively increases the apparent solubility of the cannabinoids by creating a reservoir of the drug in a more readily available form. The layered structure allows the material to have both hydrophilic and hydrophobic regions [100]. This dual nature is advantageous for its adsorption capabilities. The hydrophilic regions can interact with water molecules, while the hydrophobic regions can interact with hydrophobic compounds such as cannabinoids (CBD, CBDA, and CBC). Neusilin might also form complexes as a result of acid–base reactions, ion–dipole interactions, and hydrogen bonding [101].

The greatest results were, however, obtained for the co-dispersion delivery systems with Soluplus, which has an amphiphilic graft copolymer structure comprising three main components: polyvinyl caprolactam, polyvinyl acetate, and polyethylene glycol [102]. The polyvinyl caprolactam and polyvinyl acetate segments contribute to the polymer’s lipophilic properties, while the PEG segment imparts hydrophilicity [42]. This arrangement allows Soluplus to self-assemble into micelles when placed in an aqueous environment, effectively encapsulating hydrophobic cannabinoids within the micellar core [103]. The polyethylene glycol component in the structure also has a steric stabilizing effect on the micelles [104]. The hydrophilic segments of Soluplus might face outward, interacting with the surrounding water molecules, while the lipophilic segments interact with the cannabinoids, promoting their dispersion within the micelles. Cannabinoids might interact with Soluplus by the formation of hydrogen bonds with their hydroxyl groups [44].

In the literature, in vitro release profiles of CBD and zein and zein-WP nanoparticles in simulated gastric fluid (SGF) and simulated intestinal fluid (SIF) are provided [105]. The free CBD has low bioaccessibility, and only 29% of CBD was detected after SIF digestion. CBD, zein, and zein-WP nanoparticles showed lower sustained release during simulated gastric fluid. Pure CBD has a low solubility profile in both SIF and SGF, with less than 3% within 1 h and less than 10% of CBD released in 48 h [106]. CBD-Silica cast in PVA films show a significantly increased dissolution profile of SIF and SGF, with about 3–7% of CBD released in 1 h and about 40–45% of released CBD in 48 h. Poor solubility of CBD from hemp oil products like oral drops, capsules, and tablets in an acidic medium was also confirmed as 0% of CBD released in FaSSGF, besides one beverage enhancer [107]. Koch et al. [108] conducted a study on the dissolution properties of CBD formulations in a phosphate buffer with a pH of 6.8 and 0.5% sodium lauryl sulfate. They discovered that CBD-cyclodextrin formulations processed through freeze-drying or spray-drying, as well as CBD-mesoporous silica formulations processed through subcritical CO_2_ or atmospheric impregnation exhibited a considerable increase in their ability to dissolve in water. The study highlighted Kollidon^®^ VA64 as the excipient that displayed the greatest improvement in aqueous solubility. However, these studies are based on pure CBD, not on extracts, where there is no possible entourage effect between components of the extract. To the best of the authors’ knowledge, the release profiles for CBDA and CBC were studied for the first time.

In the current study, the differences in CBD, CBDA, and CBC profiles were mostly noticeable at the beginning of the studies, determining the speed of dissolution of cannabinoids, as well as increasing their dissolution rate, which is very important as orally administered preparations have the latest onset of action compared to other routes of administration, which can be accelerated by orally administered systems with e.g., Soluplus.

Delivery systems are also prepared to enhance the solubility and permeability of various compounds as it is presented in the literature [109,110,111]. The higher solubility of CBD and CBDA led to more efficient absorption and permeability across the gastrointestinal tract. The improved dissolution rate, increased concentration gradient, and enhanced transport contribute to the higher permeability coefficient observed in the systems compared to the pure extract in the PAMPA study. Achieving higher gastrointestinal permeability is crucial for obtaining higher oral bioavailability as it allows for more efficient absorption into the bloodstream from the gastrointestinal tract.

Oxidative stress occurs when there is an imbalance between the production of reactive oxygen species (ROS) and the body’s ability to detoxify them. While some ROS play important roles in cellular signaling and immune function, excess ROS can damage cellular components such as proteins, lipids, and DNA. Oxidative damage has been linked to several chronic diseases such as cancer, cardiovascular disease, and neurodegenerative disorders. Antioxidants neutralize harmful free radicals in the body and reduce inflammation, support the immune system, protect the brain, slow down the aging process, and improve cardiovascular health. These compounds can help protect neurons from oxidative damage, reduce inflammation, and promote cell survival, which can slow down or prevent the progression of neurodegenerative diseases. Cannabinoids exhibit various mechanisms of antioxidant properties [112]. The phenolic hydroxyl groups present in cannabinoid structures play a role in scavenging free radicals [113]. HiE antioxidant activity was slightly improved after preparing co-dispersion delivery systems; however, the changes were often not statistically significant. Similar results were observed for the inhibition of the enzymes related to neuroprotection. The increased solubility of secondary plant metabolites might have influenced their biological activity; however, this phenomenon does not always occur [114].

## 5. Conclusions

Co-dispersion delivery systems with solubilizing carriers improve the dissolution of cannabinoids: CBD, CBDA, and CBC. Particular improvement was noted for systems co-dispersed with Soluplus. It is also worth noting that the environment of the intestinal contents is the place of optimal dissolution of cannabinoids. Under these conditions, correlations between improved dissolution and better permeability of cannabinoids from co-dispersion delivery systems with solubilizing carriers were also noted. Improved dissolution of cannabinoids (CBD, CBDA, and CBC) induces better permeability through membranes simulating the walls of the digestive system as well as the blood-brain barrier, in view of their confirmed neuroprotective activity, it suggests that the developed co-dispersion delivery systems derived from *Cannabis sativa* (Henola variety) inflorescences may be valuable solutions in preventive and therapeutic procedures.

## Figures and Tables

**Table 1:** The content of cannabinoids in the prepared systems described as mg cannabinoid/g system.

System	CBD	CBDA	CBC
	mg Cannabinoid/g System		
HiE-Neusilin US2	8.73 ± 0.08	2.85 ± 0.02	0.379 ± 0.004
HiE-Soluplus	10.77 ± 0.06	3.60 ± 0.02	0.323 ± 0.004

**Table 2:** Mathematical models of release kinetics of cannabidiol in pH 1.2, pH 6.8, FaSSIF, and FeSSIF.

CBD		Mathematical Model								
		Zero-Order Kinetics		First-Order Kinetics		Higuchi Kinetics		Korsmeyer–Peppas Kinetics		
		R^2^	k	R^2^	k	R^2^	k	R^2^	k	n
pH 1.2	HiE	0.980	0.054	0.980	2.350 × 10^−4^	0.945	0.107	0.863	0.176	0.236
	HiE-Neusilin US2	0.708	0.111	0.708	4.845 × 10^−4^	0.874	0.249	0.938	0.395	0.356
	HiE-Soluplus	0.525	0.659	0.528	2.962 × 10^−3^	0.712	1.546	0.819	3.839	0.236
pH 6.8	HiE	0.985	0.075	0.985	3.269 × 10^−4^	0.975	0.151	0.959	0.194	0.332
	HiE-Neusilin US2	0.709	9.520	0.731	5.508× 10^−2^	0.871	21.254	0.948	27.542	0.443
	HiE-Soluplus	0.693	10.654	0.744	6.981 × 10^−2^	0.859	23.900	0.906	37.182	0.380
FaSSIF	HiE	0.904	2.516	0.910	1.321 × 10^−2^	0.961	5.223	0.949	17.489	0.126
	HiE-Neusilin US2	0.331	12.107	0.403	1.229 × 10^−1^	0.506	30.172	0.603	68.325	0.357
	HiE-Soluplus	0.413	8.387	0.503	1.107 × 10^−1^	0.596	20.291	0.745	73.871	0.158
FeSSIF	HiE	0.682	6.715	0.721	4.000 × 10^−2^	0.737	14.056	0.724	27.930	0.263
	HiE-Neusilin US2	0.376	16.443	0.542	5.556 × 10^−1^	0.556	40.260	0.644	89.267	0.340
	HiE-Soluplus	0.412	6.380	0.844	1.349	0.593	15.408	0.772	102.742	0.083

**Table 3:** Mathematical models of release kinetics of cannabidiolic acid in pH 1.2, pH 6.8, FaSSIF, and FeSSIF.

CBDA		Mathematical Model								
		Zero-Order Kinetics		First-Order Kinetics		Higuchi Kinetics		Korsmeyer–Peppas Kinetics		
		R^2^	k	R^2^	k	R^2^	k	R^2^	k	n
pH 1.2	HiE	0.837	0.046	0.837	1.984 × 10^−4^	0.797	0.089	0.727	0.357	0.097
	HiE-Neusilin US2	0.884	0.584	0.886	2.581 × 10^−3^	0.971	1.233	0.969	1.766	0.350
	HiE-Soluplus	0.830	0.686	0.832	3.022 × 10^−3^	0.950	1.479	0.905	1.465	0.666
pH 6.8	HiE	0.908	0.243	0.908	1.061 × 10^−3^	0.930	0.495	0.892	0.491	0.566
	HiE-Neusilin US2	0.822	13.118	0.886	1.036 × 10^−1^	0.950	28.396	0.986	46.652	0.310
	HiE-Soluplus	0.651	13.299	0.712	1.142 × 10^−1^	0.829	30.228	0.927	54.067	0.312
FaSSIF	HiE	0.907	7.126	0.927	3.942 × 10^−2^	0.986	14.965	0.994	21.800	0.328
	HiE-Neusilin US2	0.640	13.290	0.699	1.143 × 10^−1^	0.821	30.325	0.924	54.393	0.313
	HiE-Soluplus	0.649	24.294	0.753	2.473 × 10^−1^	0.823	55.120	0.908	58.247	0.647
FeSSIF	HiE	0.913	10.484	0.933	6.294 × 10^−2^	0.974	21.816	0.973	27.257	0.374
	HiE-Neusilin US2	0.579	20.645	0.822	6.125 × 10^−1^	0.768	47.898	0.885	83.584	0.344
	HiE-Soluplus	0.484	8.266	0.643	4.122 × 10^−1^	0.679	19.713	0.854	93.645	0.108

**Table 4:** Mathematical models of release kinetics of cannabichromene in pH 1.2, pH 6.8, FaSSIF, and FeSSIF.

CBC		Mathematical Model								
		Zero-Order Kinetics		First-Order Kinetics		Higuchi Kinetics		Korsmeyer–Peppas Kinetics		
		R^2^	k	R^2^	k	R^2^	k	R^2^	k	n
pH 1.2	HiE	N/D	N/D	N/D	N/D	N/D	N/D	N/D	N/D	N/D
	HiE-Neusilin US2	0.800	1.264	0.804	5.679 × 10^−3^	0.930	2.745	0.950	3.662	0.424
	HiE-Soluplus	0.825	1.340	0.830	6.056 × 10^−3^	0.943	2.885	0.954	4.227	0.370
pH 6.8	HiE	N/D	N/D	N/D	N/D	N/D	N/D	N/D	N/D	N/D
	HiE-Neusilin US2	0.642	1.810	0.651	8.519 × 10^−3^	0.808	4.091	0.863	8.712	0.265
	HiE-Soluplus	0.733	6.657	0.753	3.393 × 10^−2^	0.892	14.795	0.929	16.368	0.578
FaSSIF	HiE	0.868	5.442	0.878	2.895 × 10^−2^	0.949	11.466	0.950	18.890	0.276
	HiE-Neusilin US2	0.304	11.161	0.371	1.037 × 10^−1^	0.464	27.770	0.568	64.899	0.362
	HiE-Soluplus	0.427	5.302	0.475	8.281 × 10^−2^	0.618	12.850	0.804	76.888	0.087
FeSSIF	HiE	0.640	8.272	0.676	5.052 × 10^−2^	0.809	18.736	0.877	32.411	0.331
	HiE-Neusilin US2	0.703	17.658	0.878	3.545 × 10^−1^	0.870	39.559	0.946	78.768	0.267
	HiE-Soluplus	0.605	9.119	0.845	3.415 × 10^−1^	0.780	20.870	0.902	89.911	0.115

**Table 5:** Gastrointestinal permeability of CBD and CBDA from HiE, HiE-Neusilin US2, and HiE-Soluplus systems at pH 6.8. Results in columns with different superscript letters (a, b) differ significantly.

	P_app_ (cm/s)	
	CBD	CBDA
HiE	1.86 × 10^−7^ ± 2.24 × 10^−8 a^	7.57 × 10^−6^ ± 1.21 × 10^−7 a^
HiE-Neusilin US2	2.73 × 10^−7^ ± 9.75 × 10^−9 b^	7.56 × 10^−6^ ± 2.69 × 10^−7 a^
HiE-Soluplus	3.09 × 10^−7^ ± 1.07 × 10^−8 b^	9.51 × 10^−6^ ± 4.66 × 10^−8 b^

**Table 6:** Antioxidant activity of HiE, HiE-Neusilin US2, HiE-Soluplus in DPPH, ABTS, CUPRAC, and FRAP assay expressed as mg trolox/g plant material in the systems. Columns with different superscript letters (a, b) differ significantly.

Extract/System	DPPH	ABTS	CUPRAC	FRAP
	mg Trolox/g Plant Material			
HiE	0.85 ± 0.01 ^a^	17.04 ± 0.08 ^a^	7.03 ± 0.02 ^a^	1.58 ± 0.03 ^a^
HiE-Neusilin US2	0.86 ± 0.03 ^a^	17.30 ± 0.09 ^a^	7.65 ± 0.42 ^a^	1.56 ± 0.03 ^a^
HiE-Soluplus	0.97 ± 0.02 ^a^	18.69 ± 0.17 ^b^	7.81 ± 0.22 ^a^	1.65 ± 0.03 ^b^

**Table 7:** Inhibitory activity of HiE, HiE-Neusilin US2, HiE-Soluplus of acetylcholinesterase (presented as mg galantamine/g plant material), butyrylcholinesterase (presented as mg galantamine/g plant material), and tyrosinase (presented as mg azelaic acid/g plant material). Results with the same superscript letters in the columns are similar.

Extract/System	AChE	BChE	Tyrosinase
	mg Galantamine/g		mg Azelaic Acid/g
HiE	20.23 ± 0.43 ^a^	17.49 ± 0.16 ^a^	164.25 ± 4.44 ^a^
HiE-Neusilin US2	20.82 ± 0.44 ^a^	17.32 ± 0.24 ^a^	170.76 ± 1.86 ^a^
HiE-Soluplus	21.06 ± 0.19 ^a^	17.54 ± 0.09 ^a^	171.30 ± 2.13 ^a^

## Data Availability

Data are available in a publicly accessible repository.

## Supplementary Materials

The following supporting information can be downloaded at: https://www.mdpi.com/article/10.3390/pharmaceutics15092280/s1, Figure S1: Exemplary chromatograms of HiE-Soluplus system in apparent solubility study in pH 1.2 (a), and pH 6.8 (b); Table S1: HPLC method validation parameters.

## References

[1] Fordjour E., Manful C.F., Sey A.A., Javed R., Pham T.H., Thomas R., Cheema M. (2023). Cannabis: A Multifaceted Plant with Endless Potentials. Front. Pharmacol..

[2] Sinclair J., Collett L., Abbott J., Pate D.W., Sarris J., Armour M. (2021). Effects of Cannabis Ingestion on Endometriosis-Associated Pelvic Pain and Related Symptoms. PLoS ONE.

[3] Calapai F., Cardia L., Calapai G., Di Mauro D., Trimarchi F., Ammendolia I., Mannucci C. (2022). Effects of Cannabidiol on Locomotor Activity. Life.

[4] Anil S.M., Peeri H., Koltai H. (2022). Medical Cannabis Activity Against Inflammation: Active Compounds and Modes of Action. Front. Pharmacol..

[5] Kuhathasan N., Minuzzi L., MacKillop J., Frey B.N. (2021). The Use of Cannabinoids for Insomnia in Daily Life: Naturalistic Study. J. Med. Internet Res..

[6] Zaheer S., Kumar D., Khan M.T., Giyanwani P.R., Kiran F. (2018). Epilepsy and Cannabis: A Literature Review. Cureus.

[7] Bruni N., Della Pepa C., Oliaro-Bosso S., Pessione E., Gastaldi D., Dosio F. (2018). Cannabinoid Delivery Systems for Pain and Inflammation Treatment. Molecules.

[8] Grifoni L., Vanti G., Donato R., Sacco C., Bilia A.R. (2022). Promising Nanocarriers to Enhance Solubility and Bioavailability of Cannabidiol for a Plethora of Therapeutic Opportunities. Molecules.

[9] Gao S., Hu M. (2010). Bioavailability Challenges Associated with Development of Anti-Cancer Phenolics. Mini Rev. Med. Chem..

[10] Lucas C.J., Galettis P., Schneider J. (2018). The Pharmacokinetics and the Pharmacodynamics of Cannabinoids. Br. J. Clin. Pharmacol..

[11] Chayasirisobhon S. (2020). Mechanisms of Action and Pharmacokinetics of Cannabis. Perm. J..

[12] Subramanian N., Ghosal S.K. (2004). Enhancement of Gastrointestinal Absorption of Poorly Water Soluble Drugs via Lipid Based Systems. Indian J. Exp. Biol..

[13] Buya A.B., Beloqui A., Memvanga P.B., Préat V. (2020). Self-Nano-Emulsifying Drug-Delivery Systems: From the Development to the Current Applications and Challenges in Oral Drug Delivery. Pharmaceutics.

[14] Wang C., Wang J., Sun Y., Freeman K., Mchenry M.A., Wang C., Guo M. (2022). Enhanced Stability and Oral Bioavailability of Cannabidiol in Zein and Whey Protein Composite Nanoparticles by a Modified Anti-Solvent Approach. Foods.

[15] Hippalgaonkar K., Gul W., ElSohly M.A., Repka M.A., Majumdar S. (2011). Enhanced Solubility, Stability, and Transcorneal Permeability of Delta-8-Tetrahydrocannabinol in the Presence of Cyclodextrins. AAPS PharmSciTech.

[16] Jarho P., Pate D.W., Brenneisen R., Järvinen T. (1998). Hydroxypropyl-Beta-Cyclodextrin and Its Combination with Hydroxypropyl-Methylcellulose Increases Aqueous Solubility of Delta9-Tetrahydrocannabinol. Life Sci..

[17] Robinson D., Ritter S., Yassin M. (2022). Comparing Sublingual and Inhaled Cannabis Therapies for Low Back Pain: An Observational Open-Label Study. Rambam Maimonides Med. J..

[18] Millar S.A., Stone N.L., Yates A.S., O’Sullivan S.E. (2018). A Systematic Review on the Pharmacokinetics of Cannabidiol in Humans. Front. Pharmacol..

[19] Abu-Sawwa R., Scutt B., Park Y. (2020). Emerging Use of Epidiolex (Cannabidiol) in Epilepsy. J. Pediatr. Pharmacol. Ther..

[20] Shannon S., Lewis N., Lee H., Hughes S. (2019). Cannabidiol in Anxiety and Sleep: A Large Case Series. Perm. J..

[21] Blessing E.M., Steenkamp M.M., Manzanares J., Marmar C.R. (2015). Cannabidiol as a Potential Treatment for Anxiety Disorders. Neurotherapeutics.

[22] Wright M., Di Ciano P., Brands B. (2020). Use of Cannabidiol for the Treatment of Anxiety: A Short Synthesis of Pre-Clinical and Clinical Evidence. Cannabis Cannabinoid Res..

[23] Souza J.D.S., Zuardi A.W., Guimarães F.S., de Osório F.L., Loureiro S.R., Campos A.C., Hallak J.E.C., Dos Santos R.G., Machado Silveira I.L., Pereira-Lima K. (2022). Maintained Anxiolytic Effects of Cannabidiol after Treatment Discontinuation in Healthcare Workers during the COVID-19 Pandemic. Front. Pharmacol..

[24] Peng J., Fan M., An C., Ni F., Huang W., Luo J. (2022). A Narrative Review of Molecular Mechanism and Therapeutic Effect of Cannabidiol (CBD). Basic Clin. Pharmacol. Toxicol..

[25] Silvestro S., Mammana S., Cavalli E., Bramanti P., Mazzon E. (2019). Use of Cannabidiol in the Treatment of Epilepsy: Efficacy and Security in Clinical Trials. Molecules.

[26] Gray R.A., Whalley B.J. (2020). The Proposed Mechanisms of Action of CBD in Epilepsy. Epileptic Disord..

[27] Batalla A., Bos J., Postma A., Bossong M.G. (2021). The Impact of Cannabidiol on Human Brain Function: A Systematic Review. Front. Pharmacol..

[28] Watt G., Karl T. (2017). In Vivo Evidence for Therapeutic Properties of Cannabidiol (CBD) for Alzheimer’s Disease. Front. Pharmacol..

[29] Zou S., Kumar U. (2018). Cannabinoid Receptors and the Endocannabinoid System: Signaling and Function in the Central Nervous System. Int. J. Mol. Sci..

[30] Lu H.-C., Mackie K. (2021). Review of the Endocannabinoid System. Biol. Psychiatry Cogn. Neurosci. Neuroimaging.

[31] Simiyu D.C., Jang J.H., Lee O.R. (2022). Understanding *Cannabis sativa* L.: Current Status of Propagation, Use, Legalization, and Haploid-Inducer-Mediated Genetic Engineering. Plants.

[32] Echeverry C., Prunell G., Narbondo C., de Medina V.S., Nadal X., Reyes-Parada M., Scorza C. (2021). A Comparative In Vitro Study of the Neuroprotective Effect Induced by Cannabidiol, Cannabigerol, and Their Respective Acid Forms: Relevance of the 5-HT1A Receptors. Neurotox. Res..

[33] Goerl B., Watkins S., Metcalf C., Smith M., Beenhakker M. (2021). Cannabidiolic Acid Exhibits Entourage-like Improvements of Anticonvulsant Activity in an Acute Rat Model of Seizures. Epilepsy Res..

[34] Zagožen M., Čerenak A., Kreft S. (2021). Cannabigerol and Cannabichromene in *Cannabis sativa* L.. Acta Pharm..

[35] Shinjyo N., Di Marzo V. (2013). The Effect of Cannabichromene on Adult Neural Stem/Progenitor Cells. Neurochem. Int..

[36] Valeri A., Chiricosta L., D’Angiolini S., Pollastro F., Salamone S., Mazzon E. (2023). Cannabichromene Induces Neuronal Differentiation in NSC-34 Cells: Insights from Transcriptomic Analysis. Life.

[37] Stasiłowicz-Krzemień A., Sip S., Szulc P., Cielecka-Piontek J. (2023). Determining Antioxidant Activity of Cannabis Leaves Extracts from Different Varieties-Unveiling Nature’s Treasure Trove. Antioxidants.

[38] Pagano C., Savarese B., Coppola L., Navarra G., Avilia G., Laezza C., Bifulco M. (2023). Cannabinoids in the Modulation of Oxidative Signaling. Int. J. Mol. Sci..

[39] dos-Santos-Pereira M., Guimarães F.S., Del-Bel E., Raisman-Vozari R., Michel P.P. (2020). Cannabidiol Prevents LPS-Induced Microglial Inflammation by Inhibiting ROS/NF-ΚB-Dependent Signaling and Glucose Consumption. Glia.

[40] Pereira S.R., Hackett B., O’Driscoll D.N., Sun M.C., Downer E.J. (2021). Cannabidiol Modulation of Oxidative Stress and Signalling. Neuronal Signal..

[41] Jîtcă G., Ősz B.E., Vari C.E., Rusz C.-M., Tero-Vescan A., Pușcaș A. (2023). Cannabidiol: Bridge between Antioxidant Effect, Cellular Protection, and Cognitive and Physical Performance. Antioxidants.

[42] Sofroniou C., Baglioni M., Mamusa M., Resta C., Doutch J., Smets J., Baglioni P. (2022). Self-Assembly of Soluplus in Aqueous Solutions: Characterization and Prospectives on Perfume Encapsulation. ACS Appl. Mater. Interfaces.

[43] Al-Akayleh F., Al-Naji I., Adwan S., Al-Remawi M., Shubair M. (2022). Enhancement of Curcumin Solubility Using a Novel Solubilizing Polymer Soluplus^®^. J. Pharm. Innov..

[44] Rosiak N., Wdowiak K., Tykarska E., Cielecka-Piontek J. (2022). Amorphous Solid Dispersion of Hesperidin with Polymer Excipients for Enhanced Apparent Solubility as a More Effective Approach to the Treatment of Civilization Diseases. Int. J. Mol. Sci..

[45] Rosiak N., Tykarska E., Cielecka-Piontek J. (2023). Amorphous Pterostilbene Delivery Systems Preparation—Innovative Approach to Preparation Optimization. Pharmaceutics.

[46] Darwich M., Mohylyuk V., Kolter K., Bodmeier R., Dashevskiy A. (2023). Enhancement of Itraconazole Solubility and Release by Hot-Melt Extrusion with Soluplus^®^. J. Drug Deliv. Sci. Technol..

[47] Jha D.K., Shah D.S., Amin P.D. (2020). Thermodynamic Aspects of the Preparation of Amorphous Solid Dispersions of Naringenin with Enhanced Dissolution Rate. Int. J. Pharm..

[48] Stasiłowicz-Krzemień A., Rosiak N., Miklaszewski A., Cielecka-Piontek J. (2023). Screening of the Anti-Neurodegenerative Activity of Caffeic Acid after Introduction into Inorganic Metal Delivery Systems to Increase Its Solubility as the Result of a Mechanosynthetic Approach. Int. J. Mol. Sci..

[49] Jo K., Cho J.M., Lee H., Kim E.K., Kim H.C., Kim H., Lee J. (2019). Enhancement of Aqueous Solubility and Dissolution of Celecoxib through Phosphatidylcholine-Based Dispersion Systems Solidified with Adsorbent Carriers. Pharmaceutics.

[50] Józsa L., Nemes D., Pető Á., Kósa D., Révész R., Bácskay I., Haimhoffer Á., Vasvári G. (2023). Recent Options and Techniques to Assess Improved Bioavailability: In Vitro and Ex Vivo Methods. Pharmaceutics.

[51] Simancas-Herbada R., Fernández-Carballido A., Aparicio Blanco J., Slowing K., Rubio Retama J., López-Cabarcos E., Torres-Suarez A. (2020). Controlled Release of Highly Hydrophilic Drugs from Novel Poly(Magnesium Acrylate) Matrix Tablets. Pharmaceutics.

[52] Hens B., Tsume Y., Bermejo Sanz M., Paixao P., Koenigsknecht M., Baker J., Hasler W., Lionberger R., Fan J., Dickens J. (2017). Low Buffer Capacity and Alternating Motility Along the Human Gastrointestinal Tract: Implications for In Vivo Dissolution and Absorption of Ionizable Drugs. Mol. Pharm..

[53] Prior A., Frutos P., Correa C. Comparison of Dissolution Profiles: Current Guidelines. Proceedings of the VI Congreso SEFIG.

[54] Haimhoffer Á., Vasvári G., Budai I., Béresová M., Deák Á., Németh N., Váradi J., Sinka D., Bácskay I., Vecsernyés M. (2022). In Vitro and In Vivo Studies of a Verapamil-Containing Gastroretentive Solid Foam Capsule. Pharmaceutics.

[55] Szekalska M., Wróblewska M., Czajkowska-Kośnik A., Sosnowska K., Misiak P., Wilczewska A.Z., Winnicka K. (2023). The Spray-Dried Alginate/Gelatin Microparticles with Luliconazole as Mucoadhesive Drug Delivery System. Materials.

[56] Fischer H., Kansy M., Avdeef A., Senner F. (2007). Permeation of Permanently Positive Charged Molecules through Artificial Membranes—Influence of Physico-Chemical Properties. Eur. J. Pharm. Sci..

[57] Di L., Kerns E.H., Fan K., McConnell O.J., Carter G.T. (2003). High Throughput Artificial Membrane Permeability Assay for Blood-Brain Barrier. Eur. J. Med. Chem..

[58] Liao H., Dong W., Shi X., Liu H., Yuan K. (2012). Analysis and Comparison of the Active Components and Antioxidant Activities of Extracts from *Abelmoschus esculentus* L.. Pharmacogn. Mag..

[59] Muzykiewicz A., Florkowska K., Nowak A., Zielonka-Brzezicka J., Klimowicz A. (2019). Antioxidant Activity of St. John’s Wort Extracts Obtained with Ultrasound-Assisted Extraction. Pomeranian J. Life Sci..

[60] Stasiłowicz A., Tykarska E., Lewandowska K., Kozak M., Miklaszewski A., Kobus-Cisowska J., Szymanowska D., Plech T., Jenczyk J., Cielecka-Piontek J. (2020). Hydroxypropyl-β-Cyclodextrin as an Effective Carrier of Curcumin—Piperine Nutraceutical System with Improved Enzyme Inhibition Properties. J. Enzym. Inhib. Med. Chem..

[61] Re R., Pellegrini N., Proteggente A., Pannala A., Yang M., Rice-Evans C. (1999). Antioxidant Activity Applying an Improved ABTS Radical Cation Decolorization Assay. Free Radic. Biol. Med..

[62] Stasiłowicz-Krzemień A., Rosiak N., Płazińska A., Płaziński W., Miklaszewski A., Tykarska E., Cielecka-Piontek J. (2022). Cyclodextrin Derivatives as Promising Solubilizers to Enhance the Biological Activity of Rosmarinic Acid. Pharmaceutics.

[63] Apak R., Güçlü K., Ozyürek M., Karademir S.E., Altun M. (2005). Total Antioxidant Capacity Assay of Human Serum Using Copper(II)-Neocuproine as Chromogenic Oxidant: The CUPRAC Method. Free Radic. Res..

[64] Pohanka M. (2014). Inhibitors of Acetylcholinesterase and Butyrylcholinesterase Meet Immunity. Int. J. Mol. Sci..

[65] Chen W.-C., Tseng T.-S., Hsiao N.-W., Lin Y.-L., Wen Z.-H., Tsai C.-C., Lee Y.-C., Lin H.-H., Tsai K.-C. (2015). Discovery of Highly Potent Tyrosinase Inhibitor, T1, with Significant Anti-Melanogenesis Ability by Zebrafish in Vivo Assay and Computational Molecular Modeling. Sci. Rep..

[66] Tang G.-Y., Zhao C.-N., Xu X.-Y., Gan R.-Y., Cao S.-Y., Liu Q., Shang A., Mao Q.-Q., Li H.-B. (2019). Phytochemical Composition and Antioxidant Capacity of 30 Chinese Teas. Antioxidants.

[67] Stojkovic D., Drakulic D., Dias M.I., Zengin G., Barros L., Ivanov M., Gašic U., Rajcevic N., Stevanovic M., Ferreira I.C.F.R. (2022). *Phlomis fruticosa* L. Exerts in Vitro Antineurodegenerative and Antioxidant Activities and Induces Prooxidant Effect in Glioblastoma Cell Line. EXCLI J..

[68] Angelini P., Venanzoni R., Angeles Flores G., Tirillini B., Orlando G., Recinella L., Chiavaroli A., Brunetti L., Leone S., Di Simone S.C. (2020). Evaluation of Antioxidant, Antimicrobial and Tyrosinase Inhibitory Activities of Extracts from *Tricholosporum goniospermum*, an Edible Wild Mushroom. Antibiotics.

[69] Zhang L., Zengin G., Rocchetti G., Şenkardeş İ., Jugreet S., Mahomoodally F., Behl T., Rouphael Y., Lucini L. (2021). Phytochemical Constituents and Biological Activities of the Unexplored Plant *Rhinanthus angustifolius* subsp. *grandiflorus*. Appl. Sci..

[70] Kobenan K.C., Bini K.K.N., Kouakou M., Kouadio I.S., Zengin G., Ochou G.E.C., Boka N.R.K., Menozzi P., Ochou O.G., Dick A.E. (2021). Chemical Composition and Spectrum of Insecticidal Activity of the Essential Oils of *Ocimum gratissimum* L. and *Cymbopogon citratus* Stapf on the Main Insects of the Cotton Entomofauna in Côte d’Ivoire. Chem. Biodivers..

[71] Ferreira J., Santos S., Pereira H. (2020). In Vitro Screening for Acetylcholinesterase Inhibition and Antioxidant Activity of Quercus Suber Cork and Corkback Extracts. Evid. Based Complement. Altern. Med..

[72] Ellman G.L., Courtney K.D., Andres V., Featherstone R.M. (1961). A New and Rapid Colorimetric Determination of Acetylcholinesterase Activity. Biochem. Pharmacol..

[73] Lim T.Y., Lim Y.Y., Yule C.M. (2009). Evaluation of Antioxidant, Antibacterial and Anti-Tyrosinase Activities of Four Macaranga Species. Food Chem..

[74] Garbiec E., Rosiak N., Tykarska E., Zalewski P., Cielecka-Piontek J. (2023). Sinapic Acid Co-Amorphous Systems with Amino Acids for Improved Solubility and Antioxidant Activity. Int. J. Mol. Sci..

[75] da Costa N.F., Daniels R., Fernandes A.I., Pinto J.F. (2022). Amorphous and Co-Amorphous Olanzapine Stability in Formulations Intended for Wet Granulation and Pelletization. Int. J. Mol. Sci..

[76] Ullah M., Ullah H., Murtaza G., Mahmood Q., Hussain I. (2015). Evaluation of Influence of Various Polymers on Dissolution and Phase Behavior of Carbamazepine-Succinic Acid Cocrystal in Matrix Tablets. BioMed Res. Int..

[77] Patnaik S., Chunduri L.A.A., Akilesh M.S., Bhagavatham S.S., Kamisetti V. (2016). Enhanced Dissolution Characteristics of Piroxicam–Soluplus^®^ Nanosuspensions. J. Exp. Nanosci..

[78] Nandi U., Ajiboye A.L., Patel P., Douroumis D., Trivedi V. (2021). Preparation of Solid Dispersions of Simvastatin and Soluplus Using a Single-Step Organic Solvent-Free Supercritical Fluid Process for the Drug Solubility and Dissolution Rate Enhancement. Pharmaceuticals.

[79] Krupa A., Szlęk J., Jany B.R., Jachowicz R. (2015). Preformulation Studies on Solid Self-Emulsifying Systems in Powder Form Containing Magnesium Aluminometasilicate as Porous Carrier. AAPS PharmSciTech.

[80] Costa P., Sousa Lobo J.M. (2001). Modeling and Comparison of Dissolution Profiles. Eur. J. Pharm. Sci..

[81] Dash S., Murthy P.N., Nath L., Chowdhury P. (2010). Kinetic Modeling on Drug Release from Controlled Drug Delivery Systems. Acta Pol. Pharm..

[82] Baishya H. (2017). Application of Mathematical Models in Drug Release Kinetics of Carbidopa and Levodopa ER Tablets. J. Dev. Drugs.

[83] Andriotis E.G., Chachlioutaki K., Monou P.K., Bouropoulos N., Tzetzis D., Barmpalexis P., Chang M.-W., Ahmad Z., Fatouros D.G. (2021). Development of Water-Soluble Electrospun Fibers for the Oral Delivery of Cannabinoids. AAPS PharmSciTech.

[84] de Aguiar A.C., Vardanega R., Viganó J., Silva E.K. (2023). Supercritical Carbon Dioxide Technology for Recovering Valuable Phytochemicals from *Cannabis sativa* L. and Valorization of Its Biomass for Food Applications. Molecules.

[85] Qamar S., Manrique Y.J., Parekh H.S., Falconer J.R. (2021). Development and Optimization of Supercritical Fluid Extraction Setup Leading to Quantification of 11 Cannabinoids Derived from Medicinal Cannabis. Biology.

[86] Rochfort S., Isbel A., Ezernieks V., Elkins A., Vincent D., Deseo M.A., Spangenberg G.C. (2020). Utilisation of Design of Experiments Approach to Optimise Supercritical Fluid Extraction of Medicinal Cannabis. Sci. Rep..

[87] Tzima S., Georgiopoulou I., Louli V., Magoulas K. (2023). Recent Advances in Supercritical CO_2_ Extraction of Pigments, Lipids and Bioactive Compounds from Microalgae. Molecules.

[88] Villacís-Chiriboga J., Voorspoels S., Uyttebroek M., Ruales J., Van Camp J., Vera E., Elst K. (2021). Supercritical CO_2_ Extraction of Bioactive Compounds from Mango (*Mangifera indica* L.) Peel and Pulp. Foods.

[89] Nagybákay N.E., Syrpas M., Vilimaitė V., Tamkutė L., Pukalskas A., Venskutonis P.R., Kitrytė V. (2021). Optimized Supercritical CO_2_ Extraction Enhances the Recovery of Valuable Lipophilic Antioxidants and Other Constituents from Dual-Purpose Hop (*Humulus lupulus* L.) Variety Ella. Antioxidants.

[90] Uwineza P.A., Waśkiewicz A. (2020). Recent Advances in Supercritical Fluid Extraction of Natural Bioactive Compounds from Natural Plant Materials. Molecules.

[91] Lazarjani M.P., Young O., Kebede L., Seyfoddin A. (2021). Processing and Extraction Methods of Medicinal Cannabis: A Narrative Review. J. Cannabis Res..

[92] Safari H., Adili R., Holinstat M., Eniola-Adefeso O. (2018). Modified Two-Step Emulsion Solvent Evaporation Technique for Fabricating Biodegradable Rod-Shaped Particles in the Submicron Size Range. J. Colloid Interface Sci..

[93] Anderson L.L., Low I.K., Banister S.D., McGregor I.S., Arnold J.C. (2019). Pharmacokinetics of Phytocannabinoid Acids and Anticonvulsant Effect of Cannabidiolic Acid in a Mouse Model of Dravet Syndrome. J. Nat. Prod..

[94] Vacek J., Vostalova J., Papouskova B., Skarupova D., Kos M., Kabelac M., Storch J. (2021). Antioxidant Function of Phytocannabinoids: Molecular Basis of Their Stability and Cytoprotective Properties under UV-Irradiation. Free Radic. Biol. Med..

[95] Stella B., Baratta F., Della Pepa C., Arpicco S., Gastaldi D., Dosio F. (2021). Cannabinoid Formulations and Delivery Systems: Current and Future Options to Treat Pain. Drugs.

[96] Jeong M., Lee S., Seo C., Kwon E., Rho S., Cho M., Kim M.Y., Lee W., Lee Y.S., Hong J. (2023). Chemical Transformation of Cannabidiol into Psychotropic Cannabinoids under Acidic Reaction Conditions: Identification of Transformed Products by GC-MS. J. Food Drug Anal..

[97] Zgair A., Wong J.C., Lee J.B., Mistry J., Sivak O., Wasan K.M., Hennig I.M., Barrett D.A., Constantinescu C.S., Fischer P.M. (2016). Dietary Fats and Pharmaceutical Lipid Excipients Increase Systemic Exposure to Orally Administered Cannabis and Cannabis-Based Medicines. Am. J. Transl. Res..

[98] Mozaffari K., Willette S., Lucker B.F., Kovar S.E., Holguin F.O., Guzman I. (2021). The Effects of Food on Cannabidiol Bioaccessibility. Molecules.

[99] Park H., Cha K.-H., Hong S.H., Abuzar S.M., Lee S., Ha E.-S., Kim J.-S., Baek I.-H., Kim M.-S., Hwang S.-J. (2020). Pharmaceutical Characterization and In Vivo Evaluation of Orlistat Formulations Prepared by the Supercritical Melt-Adsorption Method Using Carbon Dioxide: Effects of Mesoporous Silica Type. Pharmaceutics.

[100] Jadhav B.V., Gawali V.B., Badadhe S.G., Bhalsing M.D. Study of Neusiln UFL2 and β-Cyclodexrtin as Solid Carriers in Solid Self-Microemulsifying Drug Delivery System of Atorvastatin Calcium Prepared by Spray Drying. https://www.asianpharmtech.com/abstract/study-of-neusiln-ufl2-and-cyclodexrtin-as-solidrncarriers-in-solid-selfmicroemulsifying-drugrndelivery-system-of-atorvas-14677.html.

[101] Azad M., Moreno J., Davé R. (2018). Stable and Fast-Dissolving Amorphous Drug Composites Preparation via Impregnation of Neusilin^®^ UFL2. J. Pharm. Sci..

[102] Cespi M., Casettari L., Palmieri G., Perinelli D., Bonacucina G. (2014). Rheological Characterization of Polyvinyl Caprolactam-Polyvinyl Acetate-Polyethylene Glycol Graft Copolymer (SoluplusA (R)) Water Dispersions. Colloid Polym. Sci..

[103] Alopaeus J.F., Hagesæther E., Tho I. (2019). Micellisation Mechanism and Behaviour of Soluplus^®^–Furosemide Micelles: Preformulation Studies of an Oral Nanocarrier-Based System. Pharmaceuticals.

[104] Piazzini V., D’Ambrosio M., Luceri C., Cinci L., Landucci E., Bilia A.R., Bergonzi M.C. (2019). Formulation of Nanomicelles to Improve the Solubility and the Oral Absorption of Silymarin. Molecules.

[105] Wang C., Cui B., Sun Y., Wang C., Guo M. (2022). Preparation, Stability, Antioxidative Property and in Vitro Release of Cannabidiol (CBD) in Zein-Whey Protein Composite Nanoparticles. LWT.

[106] Khabir Z., Partalis C., Panchal J.V., Deva A., Khatri A., Garcia-Bennett A. (2023). Enhanced Skin Penetration of Cannabidiol Using Organosilane Particles as Transdermal Delivery Vehicles. Pharmaceutics.

[107] Analakkattillam S., Langsi V.K., Hanrahan J.P., Moore E. (2021). Comparative Study of Dissolution for Cannabidiol in EU and US Hemp Oil Products by HPLC. J. Pharm. Sci..

[108] Koch N., Jennotte O., Gasparrini Y., Vandenbroucke F., Lechanteur A., Evrard B. (2020). Cannabidiol Aqueous Solubility Enhancement: Comparison of Three Amorphous Formulations Strategies Using Different Type of Polymers. Int. J. Pharm..

[109] Paczkowska-Walendowska M., Miklaszewski A., Cielecka-Piontek J. (2023). Improving Solubility and Permeability of Hesperidin through Electrospun Orange-Peel-Extract-Loaded Nanofibers. Int. J. Mol. Sci..

[110] Mahmood A., Khan L., Ijaz M., Nazir I., Naseem M., Tahir M.A., Aamir M.N., Rehman M.U., Asim M.H. (2023). Enhanced Intestinal Permeability of Cefixime by Self-Emulsifying Drug Delivery System: In-Vitro and Ex-Vivo Characterization. Molecules.

[111] Taechalertpaisarn J., Ono S., Okada O., Johnstone T.C., Lokey R.S. (2022). A New Amino Acid for Improving Permeability and Solubility in Macrocyclic Peptides through Side Chain-to-Backbone Hydrogen Bonding. J. Med. Chem..

[112] Atalay S., Jarocka-Karpowicz I., Skrzydlewska E. (2019). Antioxidative and Anti-Inflammatory Properties of Cannabidiol. Antioxidants.

[113] Zhang Y., Li H., Jin S., Lu Y., Peng Y., Zhao L., Wang X. (2022). Cannabidiol Protects against Alzheimer’s Disease in C. Elegans via ROS Scavenging Activity of Its Phenolic Hydroxyl Groups. Eur. J. Pharmacol..

[114] Paczkowska-Walendowska M., Szymanowska D., Cielecka-Piontek J. (2023). Mechanochemical Properties of Mucoadhesive Tablets Based on PVP/HPβCD Electrospun Nanofibers as Local Delivery of Polygoni Cuspidati Extract for Treating Oral Infections. Pharmaceuticals.

