# Supplementary material for: Co-Dispersion Delivery Systems with Solubilizing Carriers Improving the Solubility and Permeability of Cannabinoids (Cannabidiol, Cannabidiolic Acid, and Cannabichromene) from Cannabis sativa (Henola Variety) Inflorescences
Source: Pharmaceutics. 2023 Sep 4;15(9):2280. doi: 10.3390/pharmaceutics15092280 (PMC10537421; doi:10.3390/pharmaceutics15092280)
Supplement: Supplementary file 1 [file pharmaceutics-15-02280-s001.zip › pharmaceutics-2554592-supplementary.pdf]

Supplementary materials

Co-Dispersion Delivery Systems with Solubilizing Carriers Improving the Solubility and Permeability of Cannabinoids (Cannabidiol, Cannabidiolic Acid, and Cannabichromene) from *Cannabis sativa* (Henola Variety) Inflorescences

Anna Stasiłowicz-Krzemień [1], Piotr Szulc [2] and Judyta Cielecka-Piontek [1,3,*]

1 Department of Pharmacognosy and Biomaterials, Faculty of Pharmacy, Poznan University of Medical Sciences, Rokietnicka 3, 60-806 Poznan, Poland; astasilowicz@ump.edu.pl
2 Department of Agronomy, Poznań University of Life Sciences, Dojazd 11, 60-632 Poznan, Poland; piotr.szulc@up.poznan.pl
3 Department of Pharmacology and Phytochemistry, Institute of Natural Fibres and Medicinal Plants, Wojska Polskiego 71b, 60-630 Poznan, Poland
* Correspondence: jpiontek@ump.edu.pl

Agricultural details

The forecrop for hemp in 2022 was sugar beet. The progression of tillage procedures adhered closely to the recommended agricultural practices for this species. This regimen encompassed winter plowing on the 29th of October in 2021, a combination of harrowing and tining on the 17th of March in 2022, employment of a cultivation unit on the 6th of May in 2022, and final sowing on the 9th of May in 2022. Promptly following the sowing of hemp, which took place on the 10th of May in 2022, the herbicide Boxer 800 EC was administered at a concentration of 2.6 liters per hectare. Mineral fertilization was carried out based on the following mineral fertilizers: Lubofos 12 (200 kg/ha), potassium salt (183 kg/ha), enriched superphosphate (115 kg/ha), urea (159 kg/ha), salmag (119 kg/ha). The soil of the experimental field was classified as IIIa, complex 2. The top soil horizons, classified in terms of grain size, are deemed loamy sands, with a clay fraction accounting for 4%, dust fraction at 14%, and the sand fraction being the most substantial at 83%. The eluvial stratum features a slightly reduced clay and dust fraction, whereas the enrichment (B) and bedrock strata exhibit notably greater compactness. The pH determined in the aqueous extract expressed in pH units was 6.80. In the presence of KCl, this value displayed a decrease of approximately 0.5 units, residing within the upper range characteristic of slightly acidic conditions. The organic carbon content was about 1%, which, in terms of humus, is 1.7%. Total nitrogen content was 0.086% and the C:N ratio was about 12:1. Throughout the vegetation period, the thermal and moisture conditions demonstrated a propitious environment for the thriving growth and development of cannabis.

Results

Table S1. HPLC method validation parameters

Parameter	CBD	CBDA	CBC
calibration curve	y=10447127.56x	y=22201111.36x	y=25667184.66x
$a \pm S_a$	10447127.56 ± 31567.31	22201111.36 ± 120852.77	10447127.56 ± 238596.30
$b \pm S_b$	insignificant	insignificant	insignificant
Range of linearity (μg mL^{-1})	1 – 60	1 – 40	0.5 – 30
Correlation coefficient (r)	0.999	0.998	0.997

Limit of detection (LOD): LOD = 3 SD/a (μg mL^{-1})	0.42	0.26	0.08
Limit of quantification (LOQ): LOQ = 10 SD/a (μg mL^{-1})	1.28	0.80	0.25

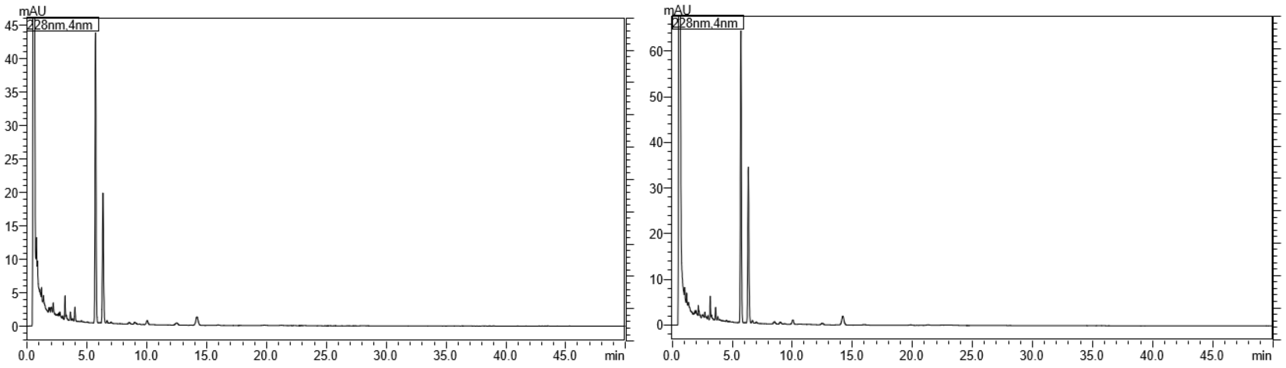

a) b)

Figure S1. Exemplary chromatograms of HiE-Soluplus system in apparent solubility study in pH 1.2 (**a**), and pH 6.8 (**b**).
